# Retrospective surveillance of viable *Bacillus cereus* group contaminations in commercial food and feed vitamin B_2_ products sold on the Belgian market using whole-genome sequencing

**DOI:** 10.3389/fmicb.2023.1173594

**Published:** 2023-06-21

**Authors:** Bert Bogaerts, Marie-Alice Fraiture, Astrid Huwaert, Tom Van Nieuwenhuysen, Bram Jacobs, Koenraad Van Hoorde, Sigrid C. J. De Keersmaecker, Nancy H. C. Roosens, Kevin Vanneste

**Affiliations:** ^1^Transversal activities in Applied Genomics, Sciensano, Brussels, Belgium; ^2^Foodborne Pathogens, Sciensano, Brussels, Belgium; ^3^Laboratory of Food Microbiology and Food Preservation, Department of Food Technology, Safety and Health, Faculty of Bioscience Engineering, Ghent University, Ghent, Belgium; ^4^Laboratory of Food and Environmental Microbiology, Earth and Life Institute, Université Catholique de Louvain, Louvain-la-Neuve, Belgium

**Keywords:** *Bacillus cereus*, food additives, riboflavin, whole-genome sequencing, surveillance

## Abstract

*Bacillus cereus* is a spore-forming bacterium that occurs as a contaminant in food and feed, occasionally resulting in food poisoning through the production of various toxins. In this study, we retrospectively characterized viable *B. cereus sensu lato* (*s.l.*) isolates originating from commercial vitamin B_2_ feed and food additives collected between 2016 and 2022 by the Belgian Federal Agency for the Safety of the Food Chain from products sold on the Belgian market. In total, 75 collected product samples were cultured on a general medium and, in case of bacterial growth, two isolates per product sample were collected and characterized using whole-genome sequencing (WGS) and subsequently characterized in terms of sequence type (ST), virulence gene profile, antimicrobial resistance (AMR) gene profile, plasmid content, and phylogenomic relationships. Viable *B. cereus* was identified in 18 of the 75 (24%) tested products, resulting in 36 WGS datasets, which were classified into eleven different STs, with ST165 (*n* = 10) and ST32 (*n* = 8) being the most common. All isolates carried multiple genes encoding virulence factors, including cytotoxin K-2 (52.78%) and cereulide (22.22%). Most isolates were predicted to be resistant to beta-lactam antibiotics (100%) and fosfomycin (88.89%), and a subset was predicted to be resistant to streptothricin (30.56%). Phylogenomic analysis revealed that some isolates obtained from different products were closely related or even identical indicating a likely common origin, whereas for some products the two isolates obtained did not show any close relationship to each other or other isolates found in other products. This study reveals that potentially pathogenic and drug-resistant *B. cereus s.l.* can be present in food and feed vitamin B_2_ additives that are commercially available, and that more research is warranted to assess whether their presence in these types of products poses a threat to consumers.

## 1. Introduction

*Bacillus cereus* is a Gram-positive bacterium that can cause food poisoning through the production of various toxins. As a common contaminant in both the food and feed production chains, *B. cereus* poses an important risk to human health and constitutes a non-negligible economic loss for the livestock sector (Bottone, 2010; Scharff, 2020; Vidic et al., 2020; Wu and Ding, 2020; Glasset et al., 2021). This bacterium belongs to the diverse *B. cereus* taxonomic group, also known as *B. cereus sensu lato* (*s.l.*), which contains other pathogenic (e.g., *Bacillus anthracis* or emetic *Bacillus mosaicus*) but also lower-risk species (e.g., *Bacillus mycoides*) (Baldwin, 2020). *B. cereus s.l.* can be classified into seven phylogenetic groups (I-VII), varying in toxin distribution, clinical significance, and risk for food poisoning (Guinebretière et al., 2010). Strains can be assigned to a phylogenetic group by comparing the sequence of the *panC* gene to a database with reference sequences (Carroll et al., 2017; Stevens et al., 2019). Nevertheless, species classification within *Bacillus cereus s.l.* has historically been challenging, and the influx of genomics data has revealed numerous inconsistencies (Carroll et al., 2022). One of the most prominent members is *B. cereus sensu stricto* (*s.s.*), colloquially known as “*B. cereus*,” which is an opportunistic pathogen that can cause foodborne disease (Guinebretière et al., 2010; Baldwin, 2020; Carroll et al., 2022). The foodborne outbreak dashboard hosted by the European Food Safety Authority (EFSA) lists 1,001 outbreaks caused by *Bacillus cereus s.l.* toxins between 2016 and 2021, associated with 11,258 human cases and 484 hospitalizations (European Food Safety Authority [EFSA], 2023). *B. cereus* strains have the ability to form spores, rendering them highly resistant to extreme environmental conditions, including heating, freezing, drying, and even radiation (Bottone, 2010). Biofilm formation can further increase their survivability and pathogenicity (Majed et al., 2016). Given the high potential of survival of its spores and its high prevalence in the food and feed chains, *B. cereus* is considered a serious “One Health” threat (Vidic et al., 2020; Cormontagne et al., 2021; Enosi Tuipulotu et al., 2021; Haque, 2021; Kalbhenn et al., 2021).

Symptoms and diseases associated with *B. cereus* infections are caused by the production of several toxins, including hemolysin BL (HBL), non-hemolytic enterotoxin (NHE) (Fox et al., 2020), and cytotoxin K (CytK) (Granum and Lund, 2006; Bottone, 2010; Fagerlund et al., 2010). Two variants of the CytK toxin have been described, CytK-1 and CytK-2, with the latter reported as being less toxic and only playing a minor role in disease (Castiaux et al., 2015; Efsa Panel on Biological Hazards [BIOHAZ], 2016; Nguyen and Tallent, 2019). Additionally, *B. cereus* strains can carry the generally plasmid-encoded *ces* operon, which produces the heat-stable emetic toxin cereulide (Kalbhenn et al., 2021). Other virulence factors of *B. cereus* that are commonly observed are metalloproteases, sphingomyelinase, and phospholipases. These enzymes can break down eukaryotic cell components such as membranes, leading to evasion of the host immune system (Guillemet et al., 2010; Oda et al., 2012; Flores-Díaz et al., 2016). Resistance to beta-lactam antibiotics through the *bla* or *bla2* gene is commonly observed in *B. cereus* and acquired resistance to numerous other antibiotics including ciprofloxacin, tetracycline, amikacin, and chloramphenicol, has also been reported (Fiedler et al., 2019). Since infections are usually self-limiting, antibiotics are rarely used to treat *B. cereus* infections (Fiedler et al., 2019; McDowell et al., 2022).

Despite the public health and economic impact on the food and feed chain, no microbiological criterion related to the presence of *B. cereus* in commercial feed products is currently established in European legislation. The only existing hygiene criterion for food products is related to dried infant formulae and dried dietary foods for medical purposes intended for infants below 6 months of age (European regulation numbers: 1831/2003, 1333/2008, 1331/2008, 2019/1381, 2073/2005, and 1441/2007). In Belgium, an action limit for *B. cereus s.l.* of 10^5^ colony-forming units/g final product (CFU/g) for food products was however, recently established by the Federal Agency for the Safety of the Food Chain (FASFC) (Federal Agency for the Safety of the Food Chain, 2018), based on EFSA recommendations (European Food Safety Authority [EFSA], 2005; Efsa Panel on Biological Hazards [BIOHAZ], 2016). Most foodborne outbreaks caused by *B. cereus s.l.* have been associated with concentrations above 10^5^ CFU/g. However, cases of both emetic and diarrheal illness caused by *B. cereus* have been reported involving concentrations ranging from 10^3^ to 10^5^ CFU/g (Efsa Panel on Biological Hazards [BIOHAZ], 2016).

Microbial fermentation products, including vitamin B_2_ (also called riboflavin), are commonly used in various industrial sectors related to food and feed production. Vitamin B_2_ is used as a feed additive to enhance animal nutrition. In the food chain, it is used as a colorant, a food additive in fortified cereal-based foods such as bread, and as a food supplement for direct consumption (Fulgoni et al., 2011; Barbau-piednoir et al., 2015; E Panel on Additives and Products or Substances used in Animal Feed [FEEDAP], Bampidis et al., 2021). Vitamin B_2_ is essential for the growth and reproduction of humans and animals (National Institutes of Health [NIH], 2022). Commercial vitamin B_2_ products are mainly produced by microbial fermentation using overproducing strains of genetically modified microorganisms (GMM) (Barbau-piednoir et al., 2015; Paracchini et al., 2017; Averianova et al., 2020; Fraiture et al., 2021). Given that the culturing conditions of microbial strains used to produce microbial fermentation products (e.g., *B. subtilis*), are usually similar to those of *B. cereus* (Deckers et al., 2020, 2022), the growth of *B. cereus* as a possible contaminant in the microbial fermentation process poses a concern for human/animal health and the economy (Averianova et al., 2020; Deckers et al., 2020, 2022). Since the conditions for vitamin production are usually favorable for bacterial growth, sterilization is advisable.

Recently, a pilot monitoring to detect GMM impurities in vitamin B_2_ products was performed in Belgium, including a viability assessment of genetically modified (GM) *Bacillus* spp. strains using classical microbiology (Fraiture et al., 2021). In the present study, several commercial vitamin B_2_ products were analyzed using classical microbiology to assess the presence of potential contaminations, followed by whole-genome sequencing (WGS), which identified the contaminants as members of the *B. cereus* group. The generated WGS data were used to characterize isolates in terms of potential virulence and antimicrobial resistance (AMR) properties and to identify putative links between isolates obtained from different products.

## 2. Materials and methods

### 2.1. Vitamin B_2_ sample collection

A total of 75 vitamin B_2_ additive products, intended for either human or animal consumption, were investigated in this study (Supplementary Table 1). These samples were collected on the Belgian market between 2016 and 2022 by the FASFC in the context of screening for the potential presence of GMMs. Metadata associated to the products was anonymized by randomly assigning lowercase letters to the product names (a-c), Greek letters to the brand names (α-β), and integers to the countries of origin (1–5). For some products, brand information was not provided but could be inferred from product names uniquely associated with a specific brand. Inferred brand codes are marked with an asterisk in the figures. The available country information can refer to the production or import origin. Consequently, the indicated countries of origin do not necessarily correspond to the country where the products were originally manufactured.

### 2.2. Bacterial isolation

At the time of sampling, the species of the potential contaminants were unknown. Therefore, a general medium was used to culture the bacteria, optimized for the detection of GMM (the initial goal of the sampling), as described elsewhere (Fraiture et al., 2021). For each sample, 1 g of the food/feed matrix was mixed with 250 ml of Brain-Heart Infusion broth (Sigma-Aldrich, Missouri, USA) for overnight aerobic incubation at 30°C (Fraiture et al., 2021). A total of 100 μl of the culture was then plated on nutrient agar (Sigma-Aldrich, Missouri, USA) for overnight aerobic incubation at 30°C. In case bacterial growth was observed, two bacterial isolates were randomly selected (not considering morphology) and subcultured for subsequent identification and characterization with whole-genome sequencing.

### 2.3. *B. cereus* enumeration

The enumeration analysis was performed directly on the product samples according to ISO 7932:2004 specifications, except for the weight of the input sample, which was decreased from 10 to 5 g. Briefly, an initial 1/10 suspension, and subsequent 1/10 serial dilutions, were made in buffered peptone water. Per suspension, 100 μL was spread on mannitol egg yolk polymyxin (MYP) agar (Oxoid, ThermoFisher Diagnostics, Erembodegem, Belgium). Plates were incubated at 30°C for 18–24 h, and if colonies were not clearly visible, incubation was continued for another 24 h. Presumptive *B. cereus* were confirmed for hemolysis and results were expressed according to ISO 7218:2007 specifications.

### 2.4. DNA extraction, DNA library preparation, and whole-genome sequencing

DNA from bacterial isolates was extracted using the NucleoSpin^®^ Food kit (MACHEREY-NAGEL, Düren, Germany), according to the manufacturer’s instructions. DNA concentrations were measured by spectrophotometry using a Nanodrop^®^ 2000 (Thermo Fisher Scientific, Waltham, MA, USA) and DNA purity was evaluated using the A260/A280 and A260/A230 ratios. Short-read DNA libraries were prepared using the Nextera XT DNA library preparation kit (Illumina, San Diego, CA, USA) according to the manufacturer’s instructions. The sequencing was carried out on an Illumina MiSeq system with the V3 chemistry, obtaining 250 bp paired-end reads, aiming for a theoretical coverage of 60x per isolate, based on the expected *Bacillus* genome size of ∼4–5 Mbp.

### 2.5. Data analysis

#### 2.5.1. Quality control and preprocessing

Raw short reads were trimmed using Trimmomatic (Bolger et al., 2014) 0.38 with the following options: “LEADING” set to 10, “TRAILING” set to 10, ‘SLIDINGWINDOW’ set to “4:20,” “MINLEN” set to 40, and “ILLUMINACLIP” set to “NexteraPE-PE.fa:2:30:10.” Processed reads were then *de novo* assembled using SPAdes (Prjibelski et al., 2020) 3.13.0 with the “--careful” option enabled and “--cov-cutoff” set to 10. Contigs smaller than 1,000 bp were removed using the seq function of Seqtk 1.3.^1^ The quality of the assemblies was evaluated using QUAST (Gurevich et al., 2013) 4.4, providing the filtered assembly as input. The percentage of reads mapping to the assembly was evaluated by mapping the processed reads against the filtered assembly using Bowtie2 (Langmead and Salzberg, 2012) 2.4.1 with the “--end-to-end” and “--sensitive” options enabled. Afterwards, the “depth” function of SAMtools (Li et al., 2009) 1.9 was used with the “-a” option enabled to calculate the median depth of the reads mapped to the assembly.

#### 2.5.2. Taxonomic identification

Kraken 2 (Wood et al., 2019) 2.0.7 was used to perform taxonomic classification of the reads using an in-house constructed database containing all NCBI RefSeq “Complete genome” entries (database accessed on the 11th of February 2021) with accession prefixes NC, NW, AC, NG, NT, NS, and NZ of the following taxonomic groups: archaea, bacteria, fungi, human, protozoa, and viruses. This database also contained a selection of animal model species reference genomes, which are listed in Supplementary Table 2. Since relying upon the NCBI taxonomy (used by Kraken 2) can be problematic for *B. cereus* because of the historical lack of a standardized nomenclature (Liu et al., 2015; Carroll et al., 2022), BTyper3 (Carroll et al., 2017) (v3.3.3) was used with the “--ani_species” and “--ani_subspecies” options enabled for species identification and *panC* clade determination, which relies on comparing the average nucleotide identity (ANI) against a curated collection of type strain genomes designed specifically for the identification of *B. cereus s.l.* (Carroll et al., 2017).

#### 2.5.3. Isolate characterization

Sequence typing was performed as described previously (Bogaerts et al., 2021), using the *B. cereus* MLST scheme retrieved from PubMLST.org (Jolley and Maiden, 2010) (accessed on the 22nd of May 2022) to assign sequence types (STs). The assembled contigs were screened for AMR genes using the NCBI Antimicrobial Resistance Gene Finder (AMRFinderPlus) tool (Feldgarden et al., 2021) 3.10.18 with the “--coverage_min” option set to 0.9, “--identity_min” set to “-1” (i.e., using the curated thresholds if they exist, 0.9 otherwise), and database version “2021-12-21.1.” The presence of genes encoding virulence factors was evaluated using a blastn-based gene detection workflow described previously (Bogaerts et al., 2021) using the gene sequences from the BTyper database (Carroll et al., 2017), for which only hits with >90% target coverage and >90% sequence identity were retained. An overview of the genes in this database is provided in Supplementary Table 3. GAMMA (Stanton et al., 2021) 1.4 was used with default options to screen the open reading frames (ORFs) for indels and premature stop codons that would result in inactive gene products. The genomic context of the AMR and virulence genes was evaluated using plasmidID 1.6.5,^2^ which performs plasmid identification based on all plasmid sequences present in RefSeq (downloaded on the 25th of April 2022). The tool was executed with the “--no-trim” option enabled, providing the trimmed forward reads, trimmed reverse reads, and assembled contigs as input. Only plasmids that were covered for >80% with >90% sequence identity were retained. The genomic context of the detected AMR and virulence genes was then predicted by cross-checking the corresponding contigs to the contigs assigned to the detected plasmids by plasmidID.

#### 2.5.4. Phylogenomic investigation

A core genome multi-locus sequence typing (cgMLST) scheme was constructed *de novo* using chewBBACA (Silva et al., 2018) 2.8.5, providing the assembled contigs as input, supplemented with all available *B. cereus* assemblies in the RefSeq database with taxonomy check status “OK” (accessed on the 30th of March 2022, *n* = 695). The scheme was constructed by using the “CreateSchema,” “AlleleCall,” and “ExtractCgMLST” commands, only retaining loci detected in ≥95% of input assemblies. The *B. cereus* s.s. reference genome (accession NZ_CP072774.1) was used as input for the creation of the required Prodigal (Hyatt et al., 2010) training file, using version 2.6.3 with default settings. A minimum spanning tree of the isolates in this study was constructed from the allele matrix using GrapeTree (Zhou et al., 2018) 2.2 with the “method” parameter set to “MSTreeV2.” The resulting phylogeny was visualized and annotated using the web-based ItoL platform (Letunic and Bork, 2021).

Separate single nucleotide polymorphism (SNP)-based phylogenies were created for each ST for which at least two isolates originated from different product samples. Reference genomes were selected from the “Complete Genomes” from the NCBI assembly database with matching ST if available and matching clonal complex otherwise (database accessed on the 30th of March 2022). STs and clonal complexes were determined using the methodology described in section “2.5.3 Isolate characterization.” When multiple genomes matched these criteria, a reference genome was randomly selected from this subset. The selected reference genomes are listed in Supplementary Table 4. Sequences annotated as plasmids were removed from the reference FASTA files prior to the analysis. SnapperDB (Dallman et al., 2017) 1.0.6 was used for calling variants and calculating SNP addresses. Each digit represents the cluster membership for the corresponding number of SNP differences, starting (right to left) with 0 (i.e., no SNP differences) to 5, 10, 25, 50, 100, and 250. Isolates sharing the same cluster digit differ by fewer than the corresponding number of SNPs to at least one other isolate in the corresponding cluster (Dallman et al., 2017). The “average_depth_cutoff” parameter was set to 25× and other settings were left at default values. SNPs located in regions annotated as phages by using the web-based PHASTER tool (Arndt et al., 2016) were removed from the VCF files before introducing them into SnapperDB. SNP matrices were extracted from SnapperDB using the “get_the_snps” function and used to construct phylogenetic trees. Maximum likelihood phylogenies were created using MEGA 10.0.4 with the “Gaps/Missing data treatment” option set to “Complete deletion,” the “Branch swap filter” set to “Very Weak,” the number of bootstrap replicates to “100,” and the “ML heuristic method” set to “SPR3.” The nucleotide substitution model was selected by model selection with the same parameters, selecting the Kimura two-parameter and the Jukes-Cantor one-parameter models, which were consequently used for the construction of the phylogenies for ST32 and ST165, and ST266, respectively.

## 3. Results

### 3.1. Overview of viable *B. cereus s.l.* contaminations in food/feed additive vitamin B_2_ samples

The potential presence of viable contaminations was investigated in a total of 75 commercial additive vitamin B_2_ products (Table 1 and Supplementary Table 1). These products were intended for either animal (*n* = 71) or human consumption (*n* = 4). All product samples were collected on the Belgian market between 2016 and 2022 by the Belgian FASFC. For each vitamin B_2_ sample, a bacterial isolation experiment was performed and when microbial growth was observed, two bacterial isolates were randomly selected and subcultured for WGS. Based on their taxonomic identification (see section “3.3 Taxonomic identification”), the presence of viable *B. cereus s.l.* was observed in 18 samples (Table 1), which was the only type of bacteria that was identified. Subsequent WGS-based characterization was consequently limited to the 36 isolates obtained from these samples. The enumeration on product samples showed that the concentration of bacteria was relatively low. The highest concentration was observed in product sample *n*°61, which had an estimated concentration of 800 CFU/g. For the remaining 17 products, the concentration was either smaller than 100 CFU/g (*n* = 15) or below the detection limit (*n* = 2). A summarized overview of all samples with viable isolates, including enumeration results, is provided in Table 1.

**TABLE 1:** Overview of food and feed vitamin B_2_ additive samples with viable *B. cereus* contaminations.

Product sample	Application	Commercial product	Brand	Sampling year	Country of origin	Product origin	Isolate name	Enumeration (CFU/g)^a^
5	Feed additive	a	α	2016	1	Producer	05-1	<1.0 × 10^2^
							05-2	
7	Feed additive	n/a	α	2016	5	Unknown	07-1	<1.0 × 10^2^
							07-2	
19	Feed additive	b	β*	2017	2	Retailer	19-1	<1.0 × 10^2^
							19-2	
26	Feed additive	n/a	α	2017	3	Producer	26-1	<1.0 × 10^2^
							26-2	
35	Feed additive	a	α*	2017	n/a	Producer	35-1	<1.0 × 10^2^
							35-2	
37	Feed additive	b	β*	2018	3	Producer	37-1	<1.0 × 10^2^
							37-2	
41	Feed additive	b	β*	2018	2	Retailer	41-1	<1.0 × 10^2^
							41-2	
42	Feed additive	b	β	2018	4	Retailer	42-1	*B. cereus* present but <4.0 × 10^2^ ^b^
							42-2	
44	Feed additive	n/a	n/a	2018	5	Producer	44-1	<1.0 × 10^2^
							44-2	
51	Feed additive	b	β*	2018	3	Producer	51-1	*B. cereus* present but <4.0 × 10^2^ ^b^
							51-2	
55	Feed additive	b	β*	2018	4	Retailer	55-1	<1.0 × 10^2^
							55-2	
59	Feed additive	n/a	β	2019	1	Producer	59-1	<1.0 × 10^2^
							59-2	
61	Feed additive	n/a	n/a	2019	4	Retailer	61-1	8.0 × 10^2^ (estimated number)
							61-2	
63	Feed additive	a	α*	2019	3	Producer	63-1	<1.0 × 10^2^
							63-2	
64	Feed additive	b	β*	2019	3	Retailer	64-1	<1.0 × 10^2^
							64-2	
71	Food additive	c	α	2021	3	Producer	71-1	<1.0 × 10^2^
							71-2	
74	Feed additive	b	β	2022	5	Producer	74-1	<1.0 × 10^2^
							74-2	
75	Feed additive	n/a	n/a	2022	3	Producer	75-1	<1.0 × 10^2^
							75-2	

^*a*^Results expressed according to ISO 7218:2007.

^*b*^The precision was too low to determine the concentration due to low colony counts.

*Inferred product brands are marked with an asterisk. Overview of the samples used in this study for which viable *B. cereus s.l.* contaminations were observed. All samples were collected on the Belgian market by the Belgian Federal Agency for the Safety of the Food Chain. If available, the field of application (food or feed additive), the commercial product name (anonymously symbolized by lowercase letters), the brand (anonymously symbolized by Greek letters), the sampling year, the origin country (anonymously symbolized by numbers), and the product origin (producer or retailer) are indicated. For each of these samples, two isolates were collected and analyzed with WGS, confirming both isolates always being identified as *B. cereus s.l.*, for which isolate names are presented in the last column. An overview of all 75 vitamin B_2_ additive products collected during this surveillance study is presented in Supplementary Table 1. n/a, not available; CFU, colony-forming unit.

### 3.2. Evaluation of sequencing quality

An overview of the generated WGS datasets and corresponding Sequence Read Archive (SRA) (Leinonen et al., 2011) accession numbers is provided in Supplementary Table 5. Raw and trimmed read counts are listed in Supplementary Table 6. The median number of read pairs before and after trimming were 692,614 and 653,622, respectively. An overview of the assembly statistics is provided in Supplementary Table 7, with a median N50 of 253,836 bp and a median total assembly length of 5,457,194 bp. The median sequencing depth ranged from 28x to 80x with a median value of 50x. The mapping rate ranged from 98.87 to 99.72%. These metrics indicate that the WGS datasets were of high quality and could be retained for further analysis.

### 3.3. Taxonomic identification

A full overview of the taxonomic identification results is provided in Supplementary Table 8. Except for isolate 61.2, for which Kraken 2 assigned 1.79% of reads to a *Bacillus* phage (NCBI taxonomy identifier: 1868826), none of the datasets contained species outside of the *Bacillus* genus with >1% associated reads. These results indicate that the isolates were not contaminated by species outside the *Bacillus* genus. All isolates were classified by BTyper3 as species within the *B. cereus* group, including *B. mosaicus panC* clade III (*n* = 33) and *Bacillus cereus s.s. panC* clade IV (*n* = 3). While historically, both taxonomic groups were sometimes referred to as *B. cereus s.s.*, recent studies have shown that *B. mosaicus* comprises a different species, spanning *panC* clades II and III (Carroll et al., 2022).

### 3.4. Isolate characterization

**FIGURE 1:**
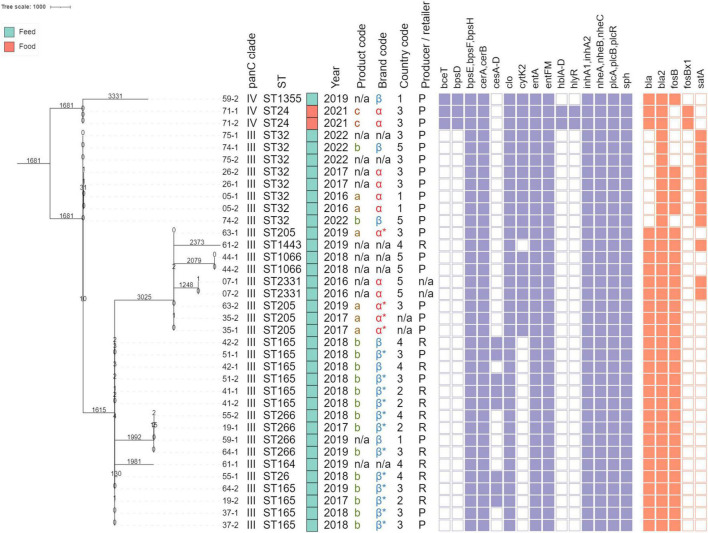
Minimum spanning tree constructed based on core genome multi-locus sequence typing (cgMLST). Branch lengths and the scale bar are expressed as number of allelic differences. The total number of loci in the cgMLST scheme was 3,500. The annotations are (from **left** to **right**): isolate name (see Table 1), *panC* clade, sequence type, product type (according to the color legend on the top left), collection year, product code, brand code, country code, producer (P) or retailer (R), detected virulence genes, and detected antimicrobial resistance (AMR) genes. Virulence genes were grouped by virulence factor (Supplementary Table 3). A filled rectangle indicates that all corresponding genes were detected with an intact open reading frame in the corresponding isolates. Brand codes inferred based on the product are marked with an asterisk. All *panC* clade III and IV isolates were identified as *B. mosaicus* and *B. cereus s.s.*, respectively. The phylogeny was rooted on the branch carrying the three *panC* clade IV isolates.

In total, eleven different sequence types were observed. The *B. mosaicus* isolates were assigned to ST265 (*n* = 10), ST32 (*n* = 8), ST205 (*n* = 4), ST266 (*n* = 4), ST1066 (*n* = 2), ST2331 (*n* = 2), ST26 (*n* = 1), ST164 (*n* = 1), and a novel ST (ST3099) similar to ST1443 (with the *pta*_348 allele instead of *pta_*5) (*n* = 1). The *B. cereus s.s.* strains were assigned to ST24 (*n* = 2) and ST1355 (*n* = 1). STs were added as annotations in Figure 1, and an overview is provided in Supplementary Table 9.

The detected virulence genes are shown in Figure 1 and are listed in Supplementary Table 10. Seventeen virulence genes were detected in all isolates, including the *bpsE*, *bpsF*, and *bpsH* genes which are part of the nine-gene *bpsX-H* operon (the other *bps* genes were absent) that is associated with the production of exo-polysaccharides; the *cerA* and *cerB* genes encoding, respectively the phospholipase CerA (cereolysin A) and sphingomyelinase C (cereolysin B); the *clo* gene encoding cereolysin O; the *entA* gene encoding enterotoxin A; the *entFM* gene encoding enterotoxin FM; the *inhA1* and *inhA2* genes encoding immune inhibitor A precursors; the *nheA-C* genes encoding the non-hemolytic enterotoxin; the *plcA* and *plcB* genes encoding phospholipase C; the *plcR* gene encoding a transcriptional regulator involved in virulence; and the *sph* gene encoding sphingomyelinase. Nineteen isolates (52.78%) additionally carried the *cytK-2* gene encoding cytotoxin K. Eight of the seventeen isolates that did not carry the *cytK-2* gene, carried the *ces* operon associated with the production of the emetic toxin cereulide. Three isolates, assigned to *Bacillus cereus s.s. panC* clade IV (ST24 and ST1355), carried the *bceT* gene encoding an enterotoxin and *bpsD* encoding an exo-polysaccharide. Lastly, these two ST24 additionally carried the complete *hblCDA* operon (including *hblB*), encoding hemolysin BL, and the *hlyR* gene encoding the hemolysin II regulator protein. None of these detected genes contained premature stop codons or frameshift mutations.

All isolates carried between two and four AMR genes, as indicated in Figure 1. A complete overview of the detected genes is provided in Supplementary Table 11. The *bla2* gene conferring resistance to beta-lactam antibiotics was observed in all isolates. Most isolates (*n* = 32, 88.89%) were also predicted to be resistant to fosfomycin since they carried the *fosB* (*n* = 30) or *fosBx1* (*n* = 2) gene. The *bla* gene conferring resistance to beta-lactams was present in 28 isolates (77.78%) isolates. Lastly, the *satA* gene, associated with resistance to streptothricin, was detected in 11 (30.56%) isolates. None of these detected genes contained premature stop codons or frameshift mutations.

Screening with plasmidID identified the presence of 27 different plasmids across the 36 isolates. An overview of the detected plasmids is provided in Supplementary Table 12. These plasmids did not carry any AMR genes, but the 273 kb NZ_CP045776.1 plasmid carried the complete *ces* operon (*cesA-D*) associated with the production of cereulide. All four genes were present in the plasmid with 100% sequence identity over their complete length. The NZ_CP045776.1 plasmid was detected in seven of the ten ST165 isolates with 100% coverage (i.e., containing the complete plasmid), all carrying the *ces* operon (Supplementary Table 10). This plasmid showed very high sequence similarity (99.97%) to the pCER270 plasmid, a highly conserved megaplasmid that carries the gene cluster for cereulide synthesis (Rasko et al., 2007). The three ST165 isolates that did not contain the plasmid also lacked the *ces* genes. Interestingly, the only other isolate that carried the *ces* genes (55-1, ST26) did not contain this plasmid or any other plasmid with the *ces* operon. Additional analyses suggested that the genes were incorporated into the chromosome of this strain (See Supplementary Figure 1). All eight isolates that carried the *ces* operon also contained the other *ces* genes (*cesH*, *cesP*, and *cesT*). Interestingly, the 05-1, 05-2, 26-1, and 26-2 isolates also carried the *cesH* gene but lacked all other *ces* genes (unpublished results).

### 3.5. Phylogenomic investigation

A phylogenetic investigation was performed to characterize the potential relationships between isolates to gain further insights into their dissemination. A cgMLST scheme was created *de novo* with chewBBACA and contained 3,500 loci. The resulting minimum spanning tree is shown in Figure 1. The *panC* groups III and IV are clearly separated into different clades of the tree and isolates with the same ST also clustered together, with larger branch lengths between isolates assigned to different STs. The two isolates originating from the single food additive intended for human consumption exhibiting microbial growth were assigned to ST24 and *panC* IV clade *B. cereus s.s.*, and were identical based on cgMLST and SNP analysis (SNP results not shown). These two isolates did not show high genomic similarity to any of the isolates collected from feed additives. Grouping isolates by ST resulted in four groups that contained at least two isolates collected from different product samples, which were further investigated by constructing SNP phylogenies and performing SNP typing.

**FIGURE 2:**
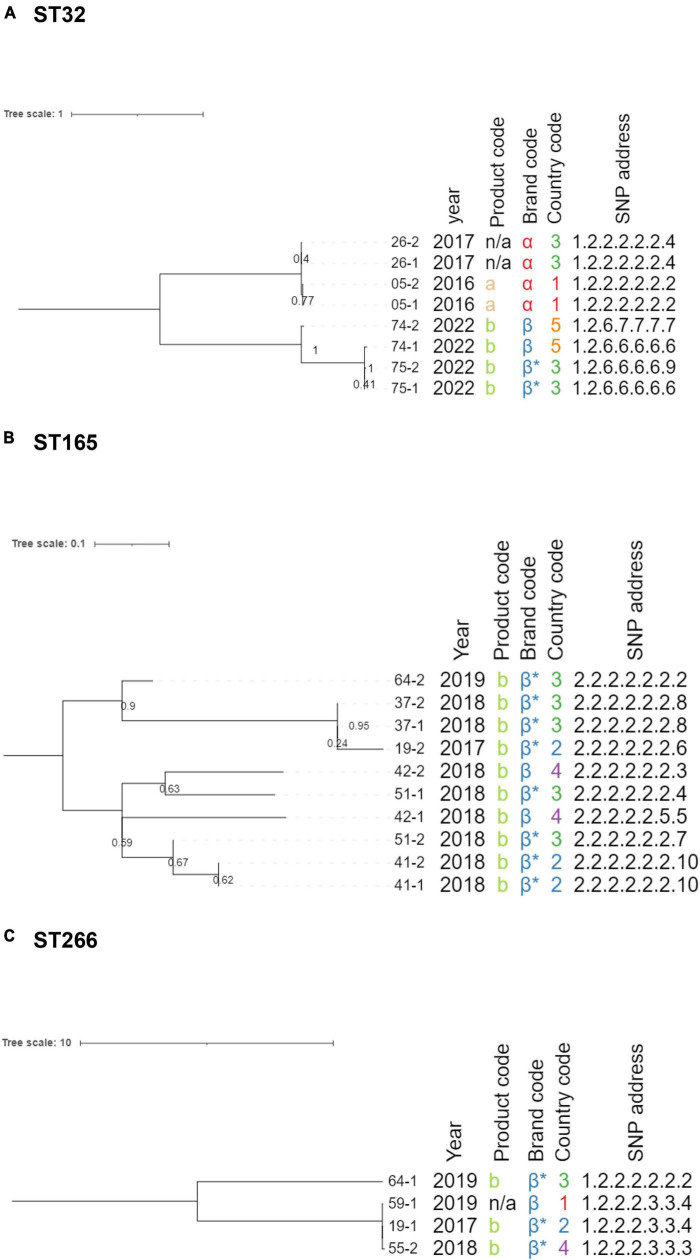
Single nucleotide polymorphism (SNP)-based maximum likelihood phylogenies for ST32 **(A)**, ST165 **(B)**, and ST266 **(C)**. Branch lengths and the scale bar are expressed as average substitutions per site. Branch labels indicate bootstrap support for the corresponding branch (as decimals). Annotations are, from left to right: isolate name, collection year, product code, brand code, country code, and SNP address. Brand codes inferred based on the product are marked with an asterisk. Midpoint rooting was applied to the phylogenetic trees.

The maximum likelihood tree for ST32 is shown in Figure 2A, and the SNP distance matrix is provided in Supplementary Table 13. The phylogeny contained eight isolates, collected from four product samples. The upper clade of the phylogeny contained samples *n*°05 and *n*°26, originating from different countries, but sold under the same brand (α) in 2016 and 2017, respectively. These four isolates were virtually identical genomically, indicating that a shared origin is likely. The lower clade of the phylogeny also contained four isolates, originating from two product samples (brand β), which were all sampled in 2022. Interestingly, the pairwise SNP distances between isolates 74-1, 75-1, and 75-2 were very small (0-1 SNP) and suggestive of a shared origin, while isolate 74-2 differed 30-31 SNPs from these isolates, despite originating from the same product sample as isolate 74-1. This suggests that at least two different *B. cereus s.l.* strains were present in the same product sample (i.e., 74-1 and 74-2), with the strain corresponding to isolate 74-1 being almost identical to those obtained in product sample *n*°75. The number of SNP differences between isolates in the upper and lower clades ranged from 66 to 90, indicating that a direct link between both clades is unlikely. Additionally, both clades had different AMR profiles, since only the isolates in the upper clade carried the *fosB* gene.

The maximum likelihood tree for ST165 is shown in Figure 2B, and the SNP distance matrix is provided in Supplementary Table 14. The phylogeny contained ten isolates collected from six brand β product samples, produced between 2017 and 2019. All isolates in this cluster were closely related to each other, with the number of pairwise SNP distances ranging from 0 to 9, indicating that a common origin is highly likely, which is remarkable given that the product samples originated from three different countries and were collected over 3 years. Despite these small genomic distances, differences were observed in the virulence gene profiles, more specifically, the *ces* operon (Supplementary Table 10), strengthening the hypothesis that these genes are plasmid-encoded, as suggested in section “3.4 Isolate characterization.” AMR gene profiles were identical for all strains assigned to this ST. Interestingly, for both product samples *n*°19 and *n*°64, the two collected isolates were assigned to different STs, including this cluster (ST165) and ST266.

Maximum likelihood tree construction for the four ST205 isolates failed because of the limited number of variable positions, but pairwise distances are shown in Supplementary Table 15. The isolates originating from the same product sample were identical, and the isolates from the two different product samples differed by three SNPs from each other. Samples *n*°35 and *n*°63 were collected from product “a” in 2017 and 2019, respectively. The low number of SNPs and identical AMR and virulence gene profiles indicate that a shared origin of the contamination in these brand α samples is highly likely, even though they were collected over a year apart.

The maximum likelihood tree for ST266 is shown in Figure 2C, and the SNP distance matrix is provided in Supplementary Table 16. The phylogeny consisted of four isolates collected from different product samples (*n*°19, *n*°55, *n*°59, and *n*°64) with different countries of origin. All four isolates had identical AMR and virulence gene profiles. The 19-1, 55-2, and 59-1 isolates were almost or completely identical, with the number of pairwise distances ranging from 0 to 2. Isolate 64-1 was more distantly related, with 24 to 27 SNPs differences compared to the other three isolates. This indicates that the aforementioned three isolates are highly likely to have a shared origin, while isolate 64-1 is potentially not directly linked.

**FIGURE 3:**
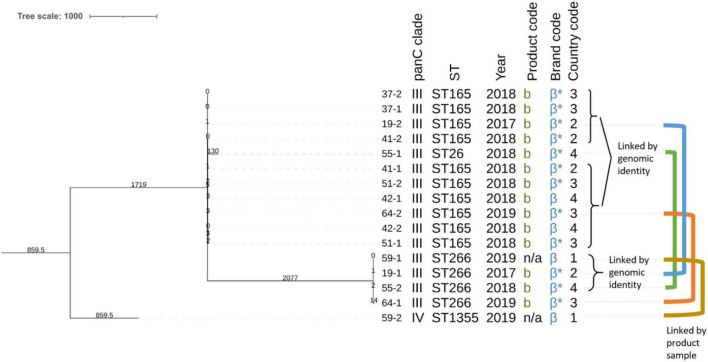
Core-genome multi-locus sequence typing (cgMLST)-based sub-phylogeny of the isolates obtained from the putative cross-contamination chain. Branch lengths and the scale bar are expressed as number of allelic differences. The total number of loci in the cgMLST scheme was 3,500. The annotations are (from **left** to **right**): isolate name (see Table 1), *panC* clade, sequence type, collection year, product code, brand code, and country code. Brand codes inferred based on the product are marked with an asterisk. The colored lines represent the isolates that are linked by their origin (i.e., isolates that were collected from the same product sample). Note that the branch lengths can differ from those observed in the phylogeny shown in Figure 1 because of the filtering of the allele matrix and the minimum spanning tree construction algorithm between the complete dataset and the partial one described in this figure. The phylogeny was rooted on the branch carrying the *panC* clade IV isolate (59-2).

Lastly, inspection of the aforementioned clusters revealed a potential “cross-contamination chain” of different strains occurring in the same product sample spanning multiple products and years (Figure 3). In this context, cross-contamination refers to the fact that *B. cereus s.l.* strains of various origins contaminate the same product sample during the manufacturing process. Within ST165, all isolates differed by at most nine SNPs from any other isolate in this cluster, suggesting that these are all variants of the same strain. Interestingly, for isolates 64-2 and 19-2 (both in the ST165 cluster), the other isolates obtained from their product samples were assigned to a different ST, ST266 (i.e., isolates 64-1 and 19-1, differing 27 SNPs from each other). Consequently, this results in a total of three strains, linked to each other by a possible cross-contamination event in two product samples. Isolate 19-1 is moreover identical to isolate 59-1 and differs a single SNP from isolate 55-2, therefore constituting the same strain. Isolate 59-2 is located in ST1355, constituting a fourth linked strain. Similarly, isolate 55-1 is located in ST26, constituting a fifth linked strain. This results in a total of five strains, linked to each other by possible cross-contamination events, covering in total eight product samples of the same brand.

## 4. Discussion

In this study, we collected viable bacterial strains from commercial vitamin B_2_ feed and food additives. Viable isolates were obtained from 18 of the 75 tested products for which always two isolates were selected for WGS, which identified all isolates as *B. cereus s.l.* and also allowed further characterization and evaluation of their phylogenomic relationships. The concentrations of the detected *B. cereus s.l.* contaminations were lower than the action limit of 10^5^ CFU/g established by the Belgian FASFC (Federal Agency for the Safety of the Food Chain, 2018). However, they may still pose a potential public health concern because of their high survivability and possibility to grow in various matrices (Guinebretière et al., 2010). Moreover, the bacterial growth level of *B. cereus* is often not correlated to its toxin-producing levels, as cereulide intoxications have previously been described for low bacterial counts because the level of cereulide production is highly variable amongst strains and matrices (Rajkovic et al., 2006; Stenfors Arnesen et al., 2008; Ellouze et al., 2021). The 36 characterized *B. cereus s.l.* isolates were assigned to eleven different sequence types (including one novel), indicating large genomic diversity. In-depth SNP analysis however, also revealed several clusters of closely related strains obtained from different product samples, suggesting a potential common origin, including isolates collected from feed additive products collected over several years.

All *B. cereus s.l.* isolates carried numerous genes encoding virulence factors, with many of them present in all isolates (e.g., *cer*, *nhe*). For the 33 *B. mosaicus* isolates assigned to *panC* clade III, virulence gene profiles were identical, except for the *ces* operon and the *cytK-2* gene, which were detected in 8 and 19 isolates, respectively. The *ces* operon is associated with emetic intoxication through the production of cereulide, and *cytK-2* encodes a diarrheal enterotoxin that may play a minor role in diarrheal disease (Fagerlund et al., 2010; Ramarao and Sanchis, 2013; Castiaux et al., 2015). Our analysis showed that the *ces* gene cluster was likely located on a plasmid closely resembling pCER270 in the seven ST165 isolates carrying these genes. All other detected virulence genes (including the *ces* genes in the ST26 isolate) were predicted to be chromosomally-encoded. However, the limited accuracy of predicting genomic context using solely short-read data should be considered for interpreting these results (Arredondo-Alonso et al., 2017). The three *B. cereus s.s.* isolates assigned to *panC* clade IV additionally carried the *bceT* and *bpsD* genes, and the two isolates assigned to ST24 also carried the *hblCDA* operon encoding hemolysin BL and the *hlyR* gene encoding a hemolysin II regulator. Because the virulence gene detection was limited to characterization at the genomic level, it could not be assessed whether these toxins are produced *in vivo* by these strains. Despite checking for the intactness of their ORFs, other elements might impact the expression of these genes and the effect of the produced proteins, and it is, therefore, challenging to determine the potential disease contribution of these putative virulence factors. It is possible to estimate the levels of some stable toxins, such as cereulide, by testing the products directly, but this method cannot be applied for enterotoxins and other less stable virulence factor proteins (Ramarao et al., 2020).

All isolates were predicted to be resistant to beta-lactam antibiotics due to the presence of the *bla* and *bla2* genes. These genes are very common in *B. cereus* (Bianco et al., 2021; Fraccalvieri et al., 2022), and resistance to these antibiotics is consequently widespread (Fiedler et al., 2019). Most isolates were also predicted to be resistant to fosfomycin due to the presence of the *fosB* (*n* = 30) and/or *fosBx1* (*n* = 2) genes, which are also widespread in *B. cereus* (Fiedler et al., 2019; Bianco et al., 2021; Fraccalvieri et al., 2022). Lastly, streptothricin resistance was predicted for eleven isolates that carried the *satA* gene. All of the detected AMR genes were predicted to be chromosomally encoded. We did not detect genes associated with resistance to other antibiotics, such as tetracycline, vancomycin, or streptogramin, as observed in *B. cereus s.l.* elsewhere (albeit more rarely) (Fiedler et al., 2019; Bianco et al., 2021; Fraccalvieri et al., 2022). Similar to the virulence genes, the characterization of AMR genes was limited to the genotypic level. Consequently, despite checking the intactness of their ORFs, this does not necessarily result in a resistant phenotype. While many studies have shown that WGS is an excellent predictor of phenotypic antibiotic resistance for numerous bacteria (Nouws et al., 2020; Bogaerts et al., 2021; Feldgarden et al., 2021), results for *B. cereus* have been mixed (Bianco et al., 2021; Mills et al., 2022). Consequently, phenotypic testing would be needed to confirm the predicted AMR profiles.

Compared to other foodborne pathogens, such as *Listeria monocytogenes* or *Salmonella* spp., the publicly available WGS data for *B. cereus s.l.* is currently limited (Carroll et al., 2019). Several studies have highlighted the added value of WGS for the detection and characterization of this pathogen (Carroll et al., 2017, 2019; Nguyen and Tallent, 2019; Bianco et al., 2021). Carroll et al. (2019) performed a retrospective WGS-based study on strains from a 2016 emetic *B. cereus* ST26 outbreak that was traced back to a fried beans manufacturer. The same ST was also observed once in this study (isolate 55-1). Interestingly, in the aforementioned study, two additional ST24 isolates were also collected from restaurants, but these were hypothesized to be unrelated to the outbreak. Our monitoring also revealed the presence of two ST24 isolates originating from the sole product intended for human consumption (product sample *n*°71). For the isolates of our study belonging to ST24 and ST26, virulence gene profiles were highly similar to the results reported by Carroll et al., including the presence of the *cytK-2* gene and the *ces* operon in the ST24 and ST26 isolates, respectively. In another recent study, WGS was used to characterize 17 *B. cereus s.l.* strains collected from human blood cultures, which were assigned to *panC* clades III and IV (Bianco et al., 2021). Nine different STs were observed, including ST24 and ST1066, which were also observed in this study with, again, highly similar virulence profiles to strains with the same ST collected in this study.

Phylogenomic investigation revealed that the studied vitamin B_2_ additives contain a diverse set of *B. cereus* isolates, as evidenced by the large phylogenomic differences and the variation in virulence and AMR genes. *B. cereus s.l.* strains were found in 18 of the 75 tested products, with five of those carrying two different strains in the same product, indicating potential cross-contamination in the manufacturing process. Several clusters of closely related isolates were identified as well, indicating a potentially shared origin. For instance, the four isolates assigned to ST266 were all collected from different product samples for which the other isolate was assigned to a different ST: ST165 (*n* = 2), ST26 (*n* = 1), and ST1355 (*n* = 1) indicating that the cross-contamination could extend to isolates assigned to these STs as well (Figure 3). The limited pairwise SNP differences between the ST165 isolates suggest a link between these isolates and the same putative cross-contamination chain. Considering that these product samples were collected over several years from brand β products with different countries of origin, a substantial cross-contamination of different *B. cereus* strains occurred. The genomic variability of the corresponding strains (i.e., the *ces* genes and predicted AMR profiles) and the geographic and temporal extent (i.e., collected between 2017-2019 from products originating from four countries) of this cross-contamination are a matter of concern. Because our analysis was limited to two isolates per tested product, we most likely did not fully capture the full variation of strains present in the products. Moreover, employed culturing conditions might have affected the strains that were collected and other contaminants may have been present that did not grow under these conditions. Further testing could therefore reveal links between products that were missed because of the sampling. Several additional clusters of closely related isolates were observed that were not part of this potential cross-contamination chain. For example, the ST32 phylogeny contained two subclades of four isolates with a very small number of pairwise SNP distances. The first clade corresponds to product samples *n*°05 and *n*°26, collected from two batches of a brand α product sampled in 2016 and 2017, originating from different countries. These findings suggest that this contamination is not sporadic but persistent, similar to the brand β cross-contamination chain. Similar results were observed for the ST32 clade (product b, brand β) and ST205 (product a, brand α). These smaller cases similarly hint at persistent contaminations in the manufacturing process of these products, most likely caused by a persistent contamination issue in and/or batch mixing by the producer.

## 5. Conclusion

This study presents the first WGS-based characterization of *B. cereus s.l.* in commercial vitamin B_2_ additives. We have shown that WGS is a powerful tool to characterize *B. cereus s.l.* strains identified in microbial fermentation products intended for the food and feed chain, by allowing complete characterization of the virulence and AMR gene profiles and investigation of phylogenomic relationships. All isolates carried numerous virulence genes and several AMR genes. Although overall large genomic diversity was observed over all isolates, several clusters of highly related isolates were found that indicated a persistent contamination issue in production chains persisting over multiple countries and years. Moreover, five instances were observed of different strains being present in the same product sample, indicating a substantial cross-contamination in the manufacturing process. These findings highlight that *B. cereus s.l.* in microbial fermentation products could be a public health concern and that a microbiological criterion for food and feed products commercialized on the European market might be required. More research and surveillance are however, needed to capture the variation of the strains present in these products, and to determine their actual public health impact. Lastly, this study raises the question whether *B. cereus s.l.* contaminations are also present in other microbial fermentation products produced by GMM and commonly used in the food and feed chains.

## Data availability statement

The datasets presented in this study can be found in online repositories. The names of the repository/repositories and accession number(s) can be found below: https://www.ncbi.nlm.nih.gov/bioproject/PRJNA903680.

## Author contributions

BB, M-AF, NR, and KeV: conceptualization. BB and M-AF: data curation and writing—original draft. BB, M-AF, AH, and TV: formal analysis. KoV and NR: funding acquisition. BB, M-AF, and AH: investigation. BB, M-AF, SD, TV, and KeV: methodology. KeV and NR: project administration. BB, M-AF, AH, TV, BJ, SD, KoV, NR, and KeV: resources. BB: software. KoV, KeV, and NR: supervision. BB, MA-F, AH, TV, BJ, KoV, SD, NR, and KeV: writing—review and editing. All authors contributed to the article and approved the submitted version.

## Footnotes

1
https://github.com/lh3/seqtk

2
https://github.com/BU-ISCIII/plasmidID
