# Supplementary material for: Retrospective surveillance of viable Bacillus cereus group contaminations in commercial food and feed vitamin B2 products sold on the Belgian market using whole-genome sequencing
Source: Front Microbiol. 2023 Jun 21;14:1173594. doi: 10.3389/fmicb.2023.1173594 (PMC10321352; doi:10.3389/fmicb.2023.1173594)
Supplement: Supplementary file 1 [file Data_Sheet_1.docx]

SUPPLEMENTARY MATERIAL

**Pilot monitoring and WGS-based characterization of viable *Bacillus cereus* contaminations in commercial food and feed vitamin B_2_ additives sold on the Belgian market**

# Figures

## Figures S1: Genomic context of the *ces* genes in isolate 55-1


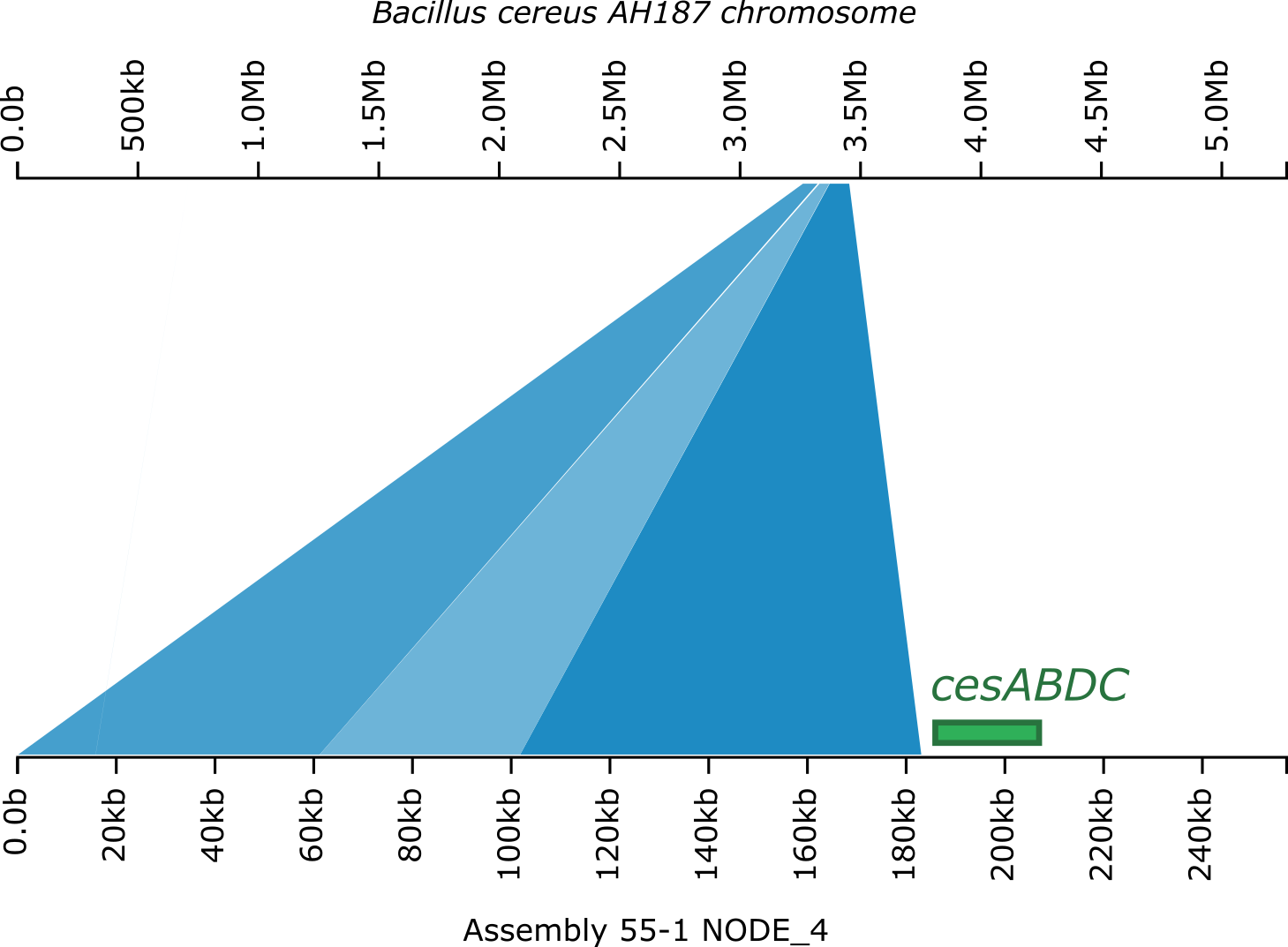


For seven of the eight isolates that carried the *cesA-D* genes, the operon was located on a contig classified as a plasmid by plasmidID. Isolate 55-1 was the exception, and additional analyses were performed to determine the genomic context of the *ces* genes in this isolate. Firstly, the contig was queried against the PLSDB [3] plasmid database, which did not return any hits. Secondly, the corresponding contig was aligned against a complete genome of the same ST (ST26): *Bacillus cereus* AH187 (RefSeq accession GCF_000021225.1) using blastn [1] 2.6.0. The resulting alignment was visualized using Kablammo [2] and shown above. The top and bottom show the chromosome of *B. cereus* AH187 and the contig carrying the *ces* genes in isolate 55-1, respectively. The blue connectors represent regions with >99% sequence identity in both sequences. The location of the *ces* genes on the isolate 55-1 contig is indicated in green. The median k-mer coverage of the corresponding contig was comparable to most other contigs in the datasets, which is typically not the case for plasmids. the median k-mer coverage of the corresponding contig was comparable to most other contigs in the datasets, which is typically not the case for plasmids (see reference below). These additional analyses support the hypothesis that these genes have been incorporated into the chromosome.

# Tables

## Table S1: Overview of the vitamin B_2_ samples

| **Sample no.** | **Application** | **Sampling year** | **Viable *B. cereus* contamination(s)** | **Product code** | **Brand code** | **Country code** |
| --- | --- | --- | --- | --- | --- | --- |
| 1 | Feed | 2016 | No | n/a | n/a | n/a |
| 2 | Feed | 2016 | No | n/a | n/a | n/a |
| 3 | Feed | 2016 | No | n/a | n/a | n/a |
| 4 | Feed | 2016 | No | n/a | n/a | n/a |
| **5** | Feed | **2016** | **Yes** | a | α | 1 |
| 6 | Feed | 2016 | No | n/a | n/a | n/a |
| **7** | Feed | **2016** | **Yes** | n/a | α | 5 |
| 8 | Feed | 2016 | No | n/a | n/a | n/a |
| 9 | Feed | 2016 | No | n/a | n/a | n/a |
| 10 | Feed | 2016 | No | n/a | n/a | n/a |
| 11 | Feed | 2016 | No | n/a | n/a | n/a |
| 12 | Feed | 2016 | No | n/a | n/a | n/a |
| 13 | Feed | 2016 | No | n/a | n/a | n/a |
| 14 | Feed | 2016 | No | n/a | n/a | n/a |
| 15 | Feed | 2016 | No | n/a | n/a | n/a |
| 16 | Feed | 2017 | No | n/a | n/a | n/a |
| 17 | Feed | 2017 | No | n/a | n/a | n/a |
| 18 | Feed | 2017 | No | n/a | n/a | n/a |
| **19** | Feed | **2017** | **Yes** | b | n/a | 2 |
| 20 | Feed | 2017 | No | n/a | n/a | n/a |
| 21 | Feed | 2017 | No | n/a | n/a | n/a |
| 22 | Feed | 2017 | No | n/a | n/a | n/a |
| 23 | Feed | 2017 | No | n/a | n/a | n/a |
| 24 | Feed | 2017 | No | n/a | n/a | n/a |
| 25 | Feed | 2017 | No | n/a | n/a | n/a |
| **26** | Feed | **2017** | **Yes** | n/a | α | 3 |
| 27 | Feed | 2017 | No | n/a | n/a | n/a |
| 28 | Feed | 2017 | No | n/a | n/a | n/a |
| 29 | Feed | 2017 | No | n/a | n/a | n/a |
| 30 | Feed | 2017 | No | n/a | n/a | n/a |
| 31 | Feed | 2017 | No | n/a | n/a | n/a |
| 32 | Feed | 2017 | No | n/a | n/a | n/a |
| 33 | Feed | 2017 | No | n/a | n/a | n/a |
| 34 | Feed | 2017 | No | n/a | n/a | n/a |
| **35** | Feed | **2017** | **Yes** | a | n/a | n/a |
| 36 | Feed | 2018 | No | n/a | n/a | n/a |
| **37** | Feed | **2018** | **Yes** | b | n/a | 3 |
| 38 | Feed | 2018 | No | n/a | n/a | n/a |
| 39 | Feed | 2018 | No | n/a | n/a | n/a |
| 40 | Feed | 2018 | No | n/a | n/a | n/a |
| **41** | Feed | **2018** | **Yes** | b | n/a | 2 |
| **42** | Feed | **2018** | **Yes** | b | β | 4 |
| 43 | Feed | 2018 | No | n/a | n/a | n/a |
| **44** | Feed | **2018** | **Yes** | n/a | n/a | 5 |
| 45 | Feed | 2018 | No | n/a | n/a | n/a |
| 46 | Feed | 2018 | No | n/a | n/a | n/a |
| 47 | Feed | 2018 | No | n/a | n/a | n/a |
| 48 | Feed | 2018 | No | n/a | n/a | n/a |
| 49 | Feed | 2018 | No | n/a | n/a | n/a |
| 50 | Feed | 2018 | No | n/a | n/a | n/a |
| **51** | Feed | **2018** | **Yes** | b | n/a | 3 |
| 52 | Feed | 2018 | No | n/a | n/a | n/a |
| 53 | Feed | 2018 | No | n/a | n/a | n/a |
| 54 | Feed | 2018 | No | n/a | n/a | n/a |
| **55** | Feed | **2018** | **Yes** | b | n/a | 4 |
| 56 | Feed | 2018 | No | n/a | n/a | n/a |
| 57 | Feed | 2018 | No | n/a | n/a | n/a |
| 58 | Feed | 2018 | No | n/a | n/a | n/a |
| **59** | Feed | **2019** | **Yes** | n/a | β | 1 |
| 60 | Feed | 2019 | No | n/a | n/a | n/a |
| **61** | Feed | **2019** | **Yes** | n/a | n/a | 4 |
| 62 | Feed | 2019 | No | n/a | n/a | n/a |
| **63** | Feed | **2019** | **Yes** | a | n/a | 3 |
| **64** | Feed | **2019** | **Yes** | b | n/a | 3 |
| 65 | Feed | 2019 | No | n/a | n/a | n/a |
| 66 | Feed | 2019 | No | n/a | n/a | n/a |
| 67 | Feed | 2019 | No | n/a | n/a | n/a |
| 68 | Food | 2020 | No | n/a | n/a | n/a |
| 69 | Feed | 2021 | No | n/a | n/a | n/a |
| 70 | Food | 2021 | No | n/a | n/a | n/a |
| **71** | Food | **2021** | **Yes** | c | α | 3 |
| 72 | Food | 2021 | No | n/a | n/a | n/a |
| 73 | Feed | 2021 | No | n/a | n/a | n/a |
| **74** | Feed | **2022** | **Yes** | b | β | 5 |
| **75** | Feed | **2022** | **Yes** | n/a | n/a | 3 |

The first, second, and third columns contain the sample number, sample origin, and sampling year, respectively. The last column indicates whether viable *B. cereus* could be isolated from the sample. Note that metadata was only requested for samples from which viable *B. cereus* could be isolated, and for some samples specific information was not provided by the original collector of the samples.

## Table S2: Mammalian reference genomes included in the Kraken 2 database

| **Species** | **Accession** |
| --- | --- |
| *Ades aegypti* | GCF_002204515 |
| *Aedes albopictus* | GCF_006496715 |
| *Anas platyrhynchos* | GCF_015476345 |
| *Stomoxys calcitrans* | GCF_001015335 |
| *Apis mellifera* | GCF_003254395 |
| *Bos taurus* | GCF_002263795 |
| *Capra hircus* | GCF_001704415 |
| *Cavia porcellus* | GCF_000151735 |
| *Chlorocebus sabaeus* | GCF_015252025 |
| *Columba livia* | GCF_000337935 |
| *Culex quinquefasciatus* | GCF_015732765 |
| *Equus caballus* | GCF_002863925 |
| *Gallus gallus* | GCF_000002315 |
| *Homo sapiens* | GCF_000001405 |
| *Ixodes scapularis* | GCF_002892825 |
| *Meleagris gallopavo* | GCF_000146605 |
| *Mesocricetus auratus* | GCF_000349665 |
| *Mus musculus* | GCF_000001635 |
| *Numida meleagris* | GCF_002078875 |
| *Ovis aries* | GCF_002742125 |
| *Rattus norvegicus* | GCF_015227675 |
| *Sus scrofa* | GCF_000003025 |

This table lists the reference genomes included in the in-house Kraken 2 database. The first column lists the species name, the second column lists the GenBank accession number.

## Table S3: Virulence genes in the BTyper database

| **Gene** | **Accession** | **description** |
| --- | --- | --- |
| *atxA* | gi_10956247 | anthrax toxin regulator |
| *bceT* | gi_984573 | enterotoxin |
| *bpsA* | NC13_RS05295 | exo-polysaccharide |
| *bpsB* | NC13_RS05300 | exo-polysaccharide |
| *bpsC* | NC13_RS05305 | exo-polysaccharide |
| *bpsD* | NC13_RS05310 | exo-polysaccharide |
| *bpsE* | NC13_RS05315 | exo-polysaccharide |
| *bpsF* | NC13_RS0532 | exo-polysaccharide |
| *bpsG* | NC13_RS05325 | exo-polysaccharide |
| *bpsH* | NC13_RS05330 | exo-polysaccharide |
| *bpsX* | NC13_RS05290 | exo-polysaccharide |
| *capA* | gi_10956390 | *B. anthracis* capsule |
| *capB* | gi_10956390 | *B. anthracis* capsule |
| *capC* | gi_10956390 | *B. anthracis* capsule |
| *capD* | gb_CP010320.1 | *B. anthracis* capsule |
| *capE* | gi_821583427 | *B. anthracis* capsule |
| *cerA* | gb_KC494692.1 | cereolysin |
| *cerB* | gb_KC527544.1 | cereolysin |
| *cesA* | gi_190015498 | emetic toxin |
| *cesB* | gi_190015498 | emetic toxin |
| *cesC* | gi_190015498 | emetic toxin |
| *cesD* | gi_190015498 | emetic toxin |
| *clo* | gb_AY818309.1 | cereolysin O |
| *cya* | gi_10956247 | *B. anthracis* pXO1-encoded toxin gene |
| *cytK_1* | gb_DQ885233.1 | cytotoxin K |
| *cytK_2* | gi_30018278 | cytotoxin K |
| *entA* | gi_30018278 | enterotoxin A |
| *entFM* | gi_30018278 | enterotoxin FM |
| *hasA* | gi_10956247 | hyaluronic acid capsule |
| *hblA* | gi_30018278 | hemolysin BL |
| *hblA* | gi_30018278 | hemolysin BL |
| *hblC* | gi_30018278 | hemolysin BL |
| *hblD* | gi_30018278 | hemolysin BL |
| *hlyII* | gi_30018278 | hemolysin II |
| *hlyIR* | gb_AY212780.1 | hemolysin II regulator |
| *inhA1* | gi_30018278 | immune inhibitor A |
| *inhA2* | gi_30018278 | immune inhibitor A |
| *lef* | gi_10956247 | *B. anthracis* pXO1-encoded toxin gene |
| *nheA* | gi_30018278 | non-hemolytic enterotoxin |
| *nheB* | gi_30018278 | non-hemolytic enterotoxin |
| *nheC* | gi_30018278 | non-hemolytic enterotoxin |
| *pagA* | gb_AF065404.1 | *B. anthracis* pXO1-encoded toxin gene |
| *plcA* | gi_30018278 | phospholipase C |
| *plcB* | gi_30018278 | phospholipase C |
| *plcR* | gi_358350621 | phospholipase C regulator |
| *plcR* | NC_003997.3 | phospholipase C regulator |
| *plcR* | NC_004722.1 | phospholipase C regulator |
| *plcR* | NC_005957.1 | phospholipase C regulator |
| *sph* | NC_003997.3 | sphingomyelinase C |

This table contains an overview of the genes in the BTyper database which was used for the detection of virulence genes. The first column lists the gene name. The second and third columns list the accession number and gene description, respectively.

## Table S4: Reference genomes used for the SNP phylogenies

| ST | NCBI assembly accession |
| --- | --- |
| ST32 | GCF_000008005.1 |
| ST165 | GCF_003812125.1 |
| ST205 | GCF_021249305.1 |
| ST266 | GCF_000013065.1 |

The first column and second column list the ST and NCBI assembly accession number of the genome used as reference genome for the SNP analysis, respectively.

## Table S5: Overview of WGS datasets

| **Isolate** | **BioSample** | **SRA accession number (raw reads)** | **Assembly accession number (contigs)** |
| --- | --- | --- | --- |
| 05-1 | SAMN31810210 | SRR22353523 | JAPNQL000000000 |
| 05-2 | SAMN31810211 | SRR22353522 | JAPNQK000000000 |
| 07-1 | SAMN31810212 | SRR22353511 | JAPNQJ000000000 |
| 07-2 | SAMN31810213 | SRR22353500 | JAPNQI000000000 |
| 19-1 | SAMN31810214 | SRR22353493 | JAPNQH000000000 |
| 19-2 | SAMN31810215 | SRR22353492 | JAPNQG000000000 |
| 26-1 | SAMN31810216 | SRR22353491 | JAPNQF000000000 |
| 26-2 | SAMN31810217 | SRR22353490 | JAPNQE000000000 |
| 35-1 | SAMN31810218 | SRR22353489 | JAPNQD000000000 |
| 35-2 | SAMN31810219 | SRR22353488 | JAPNQC000000000 |
| 37-1 | SAMN31810220 | SRR22353521 | JAPNQB000000000 |
| 37-2 | SAMN31810221 | SRR22353520 | JAPNQA000000000 |
| 41-1 | SAMN31810222 | SRR22353519 | JAPNPZ000000000 |
| 41-2 | SAMN31810223 | SRR22353518 | JAPNPY000000000 |
| 42-1 | SAMN31810224 | SRR22353517 | JAPNPX000000000 |
| 42-2 | SAMN31810225 | SRR22353516 | JAPNPW000000000 |
| 44-1 | SAMN31810226 | SRR22353515 | JAPNPV000000000 |
| 44-2 | SAMN31810227 | SRR22353514 | JAPNPU000000000 |
| 51-1 | SAMN31810228 | SRR22353513 | JAPNPT000000000 |
| 51-2 | SAMN31810229 | SRR22353512 | JAPNPS000000000 |
| 55-1 | SAMN31810230 | SRR22353510 | JAPNPR000000000 |
| 55-2 | SAMN31810231 | SRR22353509 | JAPNPQ000000000 |
| 59-1 | SAMN31810232 | SRR22353508 | JAPNPP000000000 |
| 59-2 | SAMN31810234 | SRR22353507 | JAPNPO000000000 |
| 61-1 | SAMN31810235 | SRR22353506 | JAPNPN000000000 |
| 61-2 | SAMN31810236 | SRR22353505 | JAPNPM000000000 |
| 63-1 | SAMN31810237 | SRR22353504 | JAPNPL000000000 |
| 63-2 | SAMN31810238 | SRR22353503 | JAPNPK000000000 |
| 64-1 | SAMN31810239 | SRR22353502 | JAPNPJ000000000 |
| 64-2 | SAMN31810242 | SRR22353501 | JAPNPI000000000 |
| 71-1 | SAMN31810243 | SRR22353499 | JAPNPH000000000 |
| 71-2 | SAMN31810244 | SRR22353498 | JAPNPG000000000 |
| 74-1 | SAMN31810245 | SRR22353497 | JAPNPF000000000 |
| 74-2 | SAMN31810210 | SRR22353496 | JAPNPE000000000 |
| 75-1 | SAMN31810211 | SRR22353495 | JAPNPD000000000 |
| 75-2 | SAMN31810212 | SRR22353494 | JAPNPC000000000 |

The first column lists the isolate name. The second and third columns list the SRA and assembly accession numbers, respectively.

## Table S6: Read trimming statistics

| **Isolate** | **Read pairs in** | **Read pairs after trimming** | **Forward only surviving** | **Reverse only surviving** | **Dropped pairs** |
| --- | --- | --- | --- | --- | --- |
| 05-1 | 664,643 | 626,530 | 29,474 | 4,762 | 3,877 |
| 05-2 | 760,143 | 723,623 | 29,069 | 3,882 | 3,569 |
| 07-1 | 878,761 | 765,213 | 104,847 | 1,920 | 6,781 |
| 07-2 | 956,318 | 900,578 | 45,858 | 4,028 | 5,854 |
| 19-1 | 702,134 | 672,367 | 21,243 | 5,327 | 3,197 |
| 19-2 | 915,187 | 860,592 | 44,695 | 3,831 | 6,069 |
| 26-1 | 487,110 | 459,479 | 23,476 | 1,714 | 2,441 |
| 26-2 | 549,761 | 513,688 | 30,545 | 2,352 | 3,176 |
| 35-1 | 563,780 | 531,329 | 27,523 | 2,062 | 2,866 |
| 35-2 | 618,541 | 591,998 | 21,653 | 2,420 | 2,470 |
| 37-1 | 673,693 | 649,127 | 16,964 | 3,580 | 4,022 |
| 37-2 | 742,112 | 700,603 | 30,088 | 5,804 | 5,617 |
| 41-1 | 499,903 | 470,744 | 24,440 | 2,054 | 2,665 |
| 41-2 | 814,373 | 771,129 | 33,950 | 4,047 | 5,247 |
| 42-1 | 531,138 | 507,913 | 18,466 | 2,277 | 2,482 |
| 42-2 | 801,601 | 753,184 | 39,011 | 3,532 | 5,874 |
| 44-1 | 558,309 | 517,671 | 36,285 | 1,341 | 3,012 |
| 44-2 | 760,443 | 710,279 | 41,694 | 3,377 | 5,093 |
| 51-1 | 582,406 | 547,245 | 30,127 | 2,018 | 3,016 |
| 51-2 | 813,406 | 776,900 | 27,842 | 3,270 | 5,394 |
| 55-1 | 535,557 | 497,365 | 33,593 | 1,790 | 2,809 |
| 55-2 | 577,240 | 552,132 | 20,588 | 2,052 | 2,468 |
| 59-1 | 379,409 | 356,464 | 18,255 | 2,627 | 2,063 |
| 59-2 | 757,269 | 714,620 | 36,504 | 2,629 | 3,516 |
| 61-1 | 629,451 | 600,653 | 23,596 | 2,695 | 2,507 |
| 61-2 | 694,550 | 646,188 | 40,278 | 3,425 | 4,659 |
| 63-1 | 507,098 | 477,407 | 26,062 | 1,358 | 2,271 |
| 63-2 | 1,182,740 | 1,110,655 | 56,021 | 7,178 | 8,886 |
| 64-1 | 533,935 | 508,464 | 20,876 | 2,090 | 2,505 |
| 64-2 | 875,127 | 840,679 | 26,813 | 3,361 | 4,274 |
| 71-1 | 933,798 | 880,457 | 43,558 | 3,904 | 5,879 |
| 71-2 | 698,196 | 673,738 | 19,258 | 2,020 | 3,180 |
| 74-1 | 751,208 | 727,131 | 18,377 | 2,781 | 2,919 |
| 74-2 | 720,226 | 693,735 | 21,372 | 2,011 | 3,108 |
| 75-1 | 690,678 | 658,117 | 27,425 | 1,770 | 3,366 |
| 75-2 | 640,464 | 610,896 | 25,254 | 1,457 | 2,857 |

This table lists read trimming statistics. The first column lists the isolate name. The second and third columns list the number of read pairs before and after trimming, respectively. The fourth and fifth columns list the number of forward and reverse orphaned reads after trimming, respectively. The sixth column lists the number of read pairs that were dropped.

## Table S7: *De novo* assembly statistics

| **Isolate** | **Total length** | **N50** | **Nb. of contigs** | **Median depth** | **Mapping rate (%)** |
| --- | --- | --- | --- | --- | --- |
| 05-1 | 5,435,067 | 479,346 | 46 | 47 | 99.21 |
| 05-2 | 5,431,087 | 631,859 | 33 | 60 | 99.50 |
| 07-1 | 5,846,076 | 95,356 | 40 | 54 | 99.56 |
| 07-2 | 5,417,251 | 205,709 | 35 | 68 | 99.05 |
| 19-1 | 5,620,536 | 180,606 | 138 | 49 | 99.22 |
| 19-2 | 5,556,563 | 325,247 | 90 | 62 | 98.99 |
| 26-1 | 5,465,952 | 581,335 | 99 | 35 | 99.27 |
| 26-2 | 5,472,055 | 1,150,862 | 40 | 41 | 99.23 |
| 35-1 | 5,153,384 | 147,679 | 32 | 44 | 99.30 |
| 35-2 | 5,153,606 | 265,285 | 23 | 51 | 99.72 |
| 37-1 | 5,309,222 | 200,459 | 114 | 40 | 99.25 |
| 37-2 | 5,300,401 | 248,727 | 61 | 53 | 99.01 |
| 41-1 | 5,475,552 | 212,511 | 81 | 34 | 98.98 |
| 41-2 | 5,470,736 | 139,065 | 66 | 57 | 98.92 |
| 42-1 | 5,269,428 | 525,033 | 65 | 34 | 99.45 |
| 42-2 | 5,544,410 | 531,837 | 77 | 50 | 99.02 |
| 44-1 | 5,541,200 | 248,727 | 44 | 39 | 99.27 |
| 44-2 | 5,543,114 | 248,727 | 43 | 51 | 99.37 |
| 51-1 | 5,549,098 | 154,264 | 59 | 39 | 98.87 |
| 51-2 | 5,474,076 | 246,849 | 61 | 59 | 99.41 |
| 55-1 | 5,530,798 | 247,094 | 91 | 37 | 99.26 |
| 55-2 | 5,457,194 | 195,620 | 51 | 45 | 99.55 |
| 59-1 | 5,452,037 | 380,565 | 50 | 28 | 99.06 |
| 59-2 | 5,321,748 | 383,306 | 57 | 60 | 99.58 |
| 61-1 | 5,253,220 | 1,155,406 | 40 | 48 | 99.10 |
| 61-2 | 5,449,375 | 1,170,603 | 35 | 48 | 99.46 |
| 63-1 | 5,151,509 | 246,027 | 20 | 40 | 99.27 |
| 63-2 | 5,151,646 | 228,653 | 21 | 80 | 99.47 |
| 64-1 | 5,457,269 | 253,836 | 53 | 39 | 99.11 |
| 64-2 | 5,561,020 | 222,418 | 83 | 61 | 98.92 |
| 71-1 | 5,529,837 | 376,250 | 54 | 67 | 99.63 |
| 71-2 | 5,524,149 | 359,721 | 47 | 53 | 99.64 |
| 74-1 | 5,434,014 | 359,813 | 47 | 57 | 99.56 |
| 74-2 | 5,432,342 | 373,461 | 48 | 56 | 99.57 |
| 75-1 | 5,430,628 | 479,346 | 54 | 54 | 99.63 |
| 75-2 | 5,429,501 | 631,859 | 54 | 52 | 99.66 |

The first column lists the isolate name. The second, third, and fourth column list the total length, N50, and number of contigs, respectively. These metrics were calculated by QUAST. The fifth and sixth column lists the median read depth and mapping rate, determined by mapping the processed reads against the assembly using Bowtie2.

## Table S8: Taxonomic identification results

| **Isolate** | **BTyper3 ANI species** | **Kraken 2 classification (species-level)** |
| --- | --- | --- |
| 05-1 | *B. mosaicus* | Bacillus cereus (64.85), Bacillus thuringiensis (3.56), Bacillus mycoides (1.69), Bacillus anthracis (1.16) |
| 05-2 | *B. mosaicus* | Bacillus cereus (69.98), Bacillus thuringiensis (3.41), Bacillus mycoides (1.53) |
| 07-1 | *B. mosaicus* | Bacillus cereus (18.88), Bacillus thuringiensis (7.49), Bacillus paranthracis (7.19), Bacillus mycoides (3.26), Bacillus anthracis (2.28), Bacillus luti (1.87), Bacillus albus (1.72), Bacillus mobilis (1.60), Bacillus wiedmannii (1.53), Bacillus pseudomycoides (1.02) |
| 07-2 | *B. mosaicus* | Bacillus cereus (20.58), Bacillus thuringiensis (8.20), Bacillus paranthracis (7.31), Bacillus mycoides (3.39), Bacillus anthracis (2.35), Bacillus luti (1.90), Bacillus albus (1.86), Bacillus mobilis (1.63), Bacillus wiedmannii (1.56), Bacillus pseudomycoides (1.13) |
| 19-1 | *B. mosaicus* | Bacillus cereus (29.27), Bacillus thuringiensis (6.49), Bacillus mycoides (3.13), Bacillus anthracis (2.36), Bacillus luti (1.85), Bacillus paranthracis (1.67), Bacillus albus (1.61), Bacillus mobilis (1.60), Bacillus wiedmannii (1.47), Bacillus pseudomycoides (1.08) |
| 19-2 | *B. mosaicus* | Bacillus cereus (11.92), Bacillus thuringiensis (8.55), Bacillus paranthracis (4.05), Bacillus mycoides (3.57), Bacillus anthracis (2.43), Bacillus luti (2.03), Bacillus mobilis (1.77), Bacillus albus (1.74), Bacillus wiedmannii (1.61), Bacillus pseudomycoides (1.06), Bacillus cytotoxicus (1.04), Bacillus sp. BD59S (1.01) |
| 26-1 | *B. mosaicus* | Bacillus cereus (65.05), Bacillus thuringiensis (3.64), Bacillus mycoides (1.67), Bacillus anthracis (1.20), Bacillus paranthracis (1.14) |
| 26-2 | *B. mosaicus* | Bacillus cereus (66.48), Bacillus thuringiensis (3.57), Bacillus mycoides (1.58), Bacillus paranthracis (1.18) |
| 35-1 | *B. mosaicus* | Bacillus cereus (19.01), Bacillus thuringiensis (8.20), Bacillus paranthracis (7.85), Bacillus mycoides (3.65), Bacillus anthracis (2.93), Bacillus luti (2.23), Bacillus mobilis (1.94), Bacillus albus (1.91), Bacillus wiedmannii (1.71), Bacillus pseudomycoides (1.29), Bacillus cytotoxicus (1.11) |
| 35-2 | *B. mosaicus* | Bacillus cereus (19.80), Bacillus thuringiensis (8.56), Bacillus paranthracis (8.14), Bacillus mycoides (3.69), Bacillus anthracis (2.99), Bacillus luti (2.20), Bacillus albus (1.97), Bacillus mobilis (1.97), Bacillus wiedmannii (1.74), Bacillus pseudomycoides (1.17), Bacillus cytotoxicus (1.04) |
| 37-1 | *B. mosaicus* | Bacillus cereus (10.69), Bacillus thuringiensis (7.85), Bacillus paranthracis (4.35), Bacillus mycoides (3.33), Bacillus anthracis (2.43), Bacillus luti (1.76), Bacillus albus (1.57), Bacillus mobilis (1.56), Bacillus wiedmannii (1.42), Bacillus sp. BD59S (1.03), Bacillus cytotoxicus (1.00) |
| 37-2 | *B. mosaicus* | Bacillus cereus (11.62), Bacillus thuringiensis (8.18), Bacillus paranthracis (3.78), Bacillus mycoides (3.73), Bacillus anthracis (2.55), Bacillus luti (2.17), Bacillus mobilis (1.84), Bacillus albus (1.81), Bacillus wiedmannii (1.72), Bacillus pseudomycoides (1.10), Bacillus sp. BD59S (1.07), Bacillus cytotoxicus (1.06) |
| 41-1 | *B. mosaicus* | Bacillus cereus (12.72), Bacillus thuringiensis (9.29), Bacillus mycoides (3.68), Bacillus paranthracis (3.46), Bacillus anthracis (2.63), Bacillus luti (2.07), Bacillus mobilis (1.79), Bacillus albus (1.78), Bacillus wiedmannii (1.65), Bacillus cytotoxicus (1.24), Bacillus pseudomycoides (1.11), Bacillus sp. BD59S (1.05) |
| 41-2 | *B. mosaicus* | Bacillus cereus (11.96), Bacillus thuringiensis (8.27), Bacillus paranthracis (3.72), Bacillus mycoides (3.59), Bacillus anthracis (2.43), Bacillus luti (2.10), Bacillus albus (1.79), Bacillus mobilis (1.79), Bacillus wiedmannii (1.65), Bacillus cytotoxicus (1.07), Bacillus pseudomycoides (1.06), Bacillus sp. BD59S (1.01) |
| 42-1 | *B. mosaicus* | Bacillus cereus (12.07), Bacillus thuringiensis (9.41), Bacillus mycoides (3.50), Bacillus paranthracis (3.50), Bacillus anthracis (2.52), Bacillus luti (1.93), Bacillus mobilis (1.69), Bacillus albus (1.65), Bacillus wiedmannii (1.53), Bacillus cytotoxicus (1.41), Bacillus pseudomycoides (1.07), Bacillus sp. BD59S (1.00) |
| 42-2 | *B. mosaicus* | Bacillus cereus (11.35), Bacillus thuringiensis (8.34), Bacillus paranthracis (3.61), Bacillus mycoides (3.36), Bacillus anthracis (2.27), Bacillus luti (1.87), Bacillus mobilis (1.64), Bacillus albus (1.61), Bacillus wiedmannii (1.48), Bacillus cytotoxicus (1.07), Bacillus pseudomycoides (1.01) |
| 44-1 | *B. mosaicus* | Bacillus cereus (23.46), Bacillus thuringiensis (9.16), Bacillus paranthracis (6.82), Bacillus mycoides (3.51), Bacillus anthracis (2.98), Bacillus luti (2.03), Bacillus mobilis (1.78), Bacillus albus (1.73), Bacillus wiedmannii (1.65), Bacillus pseudomycoides (1.27), Bacillus cytotoxicus (1.04) |
| 44-2 | *B. mosaicus* | Bacillus cereus (21.55), Bacillus thuringiensis (9.07), Bacillus paranthracis (6.04), Bacillus mycoides (3.30), Bacillus anthracis (2.60), Bacillus luti (1.94), Bacillus mobilis (1.75), Bacillus albus (1.63), Bacillus wiedmannii (1.61), Bacillus pseudomycoides (1.13), Bacillus cytotoxicus (1.02) |
| 51-1 | *B. mosaicus* | Bacillus cereus (13.02), Bacillus thuringiensis (10.02), Bacillus mycoides (3.74), Bacillus paranthracis (3.48), Bacillus anthracis (2.64), Bacillus luti (2.05), Bacillus albus (1.79), Bacillus mobilis (1.75), Bacillus wiedmannii (1.60), Bacillus cytotoxicus (1.27), Bacillus pseudomycoides (1.08) |
| 51-2 | *B. mosaicus* | Bacillus cereus (12.54), Bacillus thuringiensis (8.74), Bacillus paranthracis (3.99), Bacillus mycoides (3.76), Bacillus anthracis (2.55), Bacillus luti (2.14), Bacillus mobilis (1.87), Bacillus albus (1.87), Bacillus wiedmannii (1.69), Bacillus cytotoxicus (1.10), Bacillus pseudomycoides (1.10), Bacillus sp. BD59S (1.03) |
| 55-1 | *B. mosaicus* | Bacillus cereus (12.75), Bacillus thuringiensis (9.53), Bacillus paranthracis (4.11), Bacillus mycoides (3.86), Bacillus anthracis (2.70), Bacillus luti (2.16), Bacillus mobilis (1.94), Bacillus albus (1.85), Bacillus wiedmannii (1.70), Bacillus sp. FDAARGOS_527 (1.46), Bacillus cytotoxicus (1.17), Bacillus pseudomycoides (1.15), Bacillus sp. BD59S (1.06) |
| 55-2 | *B. mosaicus* | Bacillus cereus (31.46), Bacillus thuringiensis (6.76), Bacillus mycoides (3.20), Bacillus anthracis (2.37), Bacillus luti (1.94), Bacillus paranthracis (1.76), Bacillus mobilis (1.63), Bacillus albus (1.60), Bacillus wiedmannii (1.53), Bacillus pseudomycoides (1.04) |
| 59-1 | *B. mosaicus* | Bacillus cereus (32.77), Bacillus thuringiensis (6.75), Bacillus mycoides (3.27), Bacillus anthracis (2.48), Bacillus luti (1.94), Bacillus paranthracis (1.78), Bacillus mobilis (1.68), Bacillus albus (1.64), Bacillus wiedmannii (1.53), Bacillus pseudomycoides (1.08) |
| 59-2 | *B. cereus s.s.* | Bacillus thuringiensis (17.30), Bacillus cereus (17.03), Bacillus mycoides (4.18), Bacillus luti (2.36), Bacillus albus (1.37), Bacillus pseudomycoides (1.23), Bacillus wiedmannii (1.15), Bacillus mobilis (1.15), Bacillus anthracis (1.14), Bacillus cytotoxicus (1.11), Bacillus bombysepticus (1.09) |
| 61-1 | *B. mosaicus* | Bacillus cereus (20.56), Bacillus thuringiensis (8.77), Bacillus mycoides (3.74), Bacillus anthracis (3.07), Bacillus paranthracis (3.00), Bacillus luti (2.49), Bacillus mobilis (1.96), Bacillus albus (1.94), Bacillus wiedmannii (1.80), Bacillus pseudomycoides (1.30), Bacillus cytotoxicus (1.20), Bacillus sp. BD59S (1.02) |
| 61-2 | *B. mosaicus* | Bacillus cereus (22.07), Bacillus thuringiensis (7.46), Bacillus paranthracis (6.39), Bacillus mycoides (3.57), Bacillus anthracis (2.65), Bacillus luti (2.09), Bacillus mobilis (1.85), Bacillus phage vB_BtS_BMBtp14 (1.79), Bacillus albus (1.74), Bacillus wiedmannii (1.71), Bacillus pseudomycoides (1.17), Bacillus cytotoxicus (1.07) |
| 63-1 | *B. mosaicus* | Bacillus cereus (19.86), Bacillus thuringiensis (8.48), Bacillus paranthracis (8.23), Bacillus mycoides (3.78), Bacillus anthracis (3.00), Bacillus luti (2.23), Bacillus albus (1.96), Bacillus mobilis (1.95), Bacillus wiedmannii (1.75), Bacillus pseudomycoides (1.29), Bacillus cytotoxicus (1.12) |
| 63-2 | *B. mosaicus* | Bacillus cereus (18.60), Bacillus thuringiensis (8.20), Bacillus paranthracis (6.90), Bacillus mycoides (3.24), Bacillus anthracis (2.58), Bacillus luti (1.89), Bacillus albus (1.78), Bacillus mobilis (1.75), Bacillus wiedmannii (1.50) |
| 64-1 | *B. mosaicus* | Bacillus cereus (31.62), Bacillus thuringiensis (6.67), Bacillus mycoides (3.24), Bacillus anthracis (2.48), Bacillus luti (1.96), Bacillus paranthracis (1.77), Bacillus albus (1.65), Bacillus mobilis (1.63), Bacillus wiedmannii (1.51), Bacillus pseudomycoides (1.09) |
| 64-2 | *B. mosaicus* | Bacillus cereus (12.58), Bacillus thuringiensis (9.28), Bacillus paranthracis (4.30), Bacillus mycoides (3.70), Bacillus anthracis (2.47), Bacillus luti (2.07), Bacillus mobilis (1.80), Bacillus albus (1.78), Bacillus wiedmannii (1.62), Bacillus cytotoxicus (1.07), Bacillus pseudomycoides (1.06), Bacillus sp. BD59S (1.01) |
| 71-1 | *B. cereus s.s.* | Bacillus cereus (25.72), Bacillus thuringiensis (11.20), Bacillus mycoides (3.44), Bacillus luti (1.73), Bacillus anthracis (1.10), Bacillus pseudomycoides (1.09), Bacillus cytotoxicus (1.07) |
| 71-2 | *B. cereus s.s.* | Bacillus cereus (25.95), Bacillus thuringiensis (11.50), Bacillus mycoides (3.54), Bacillus luti (1.84), Bacillus pseudomycoides (1.08), Bacillus cytotoxicus (1.04), Bacillus anthracis (1.04), Bacillus albus (1.02), Bacillus mobilis (1.00), Bacillus wiedmannii (1.00) |
| 74-1 | *B. mosaicus* | Bacillus cereus (64.17), Bacillus thuringiensis (6.64), Bacillus mycoides (1.56) |
| 74-2 | *B. mosaicus* | Bacillus cereus (66.86), Bacillus thuringiensis (3.53), Bacillus mycoides (1.67) |
| 75-1 | *B. mosaicus* | Bacillus cereus (67.00), Bacillus thuringiensis (3.87), Bacillus mycoides (1.60) |
| 75-2 | *B. mosaicus* | Bacillus cereus (68.05), Bacillus thuringiensis (3.85), Bacillus mycoides (1.60) |

The first column lists the isolate name. The second column lists the species detected by the BTyper ANI classification. The third column lists the species level classification of the reads by Kraken 2, the number between brackets corresponds to the percentage of reads classified to the corresponding species. Unclassified reads were not included.

## Table S9: *B. cereus* MLST sequence types

| **Isolate** | **ST** | **Clonal complex** |
| --- | --- | --- |
| 05-1 | 32 | - |
| 05-2 | 32 | - |
| 07-1 | 2331 | ST-205 complex |
| 07-2 | 2331 | ST-205 complex |
| 19-1 | 266 | - |
| 19-2 | 165 | - |
| 26-1 | 32 | - |
| 26-2 | 32 | - |
| 35-1 | 205 | ST-205 complex |
| 35-2 | 205 | ST-205 complex |
| 37-1 | 165 | - |
| 37-2 | 165 | - |
| 41-1 | 165 | - |
| 41-2 | 165 | - |
| 42-1 | 165 | - |
| 42-2 | 165 | - |
| 44-1 | 1066 | ST-205 complex |
| 44-2 | 1066 | ST-205 complex |
| 51-1 | 165 | - |
| 51-2 | 165 | - |
| 55-1 | 26 | - |
| 55-2 | 266 | - |
| 59-1 | 266 | - |
| 59-2 | 1355 | ST-18 complex |
| 61-1 | 164 | - |
| 61-2 | 1443 | ST-205 complex |
| 63-1 | 205 | ST-205 complex |
| 63-2 | 205 | ST-205 complex |
| 64-1 | 266 | - |
| 64-2 | 165 | - |
| 71-1 | 24 | - |
| 71-2 | 24 | - |
| 74-1 | 32 | - |
| 74-2 | 32 | - |
| 75-1 | 32 | - |
| 75-2 | 32 | - |

The first column lists the isolate name, the second and third columns list the sequence type and clonal complex, respectively. Only perfect matches were considered (i.e., all seven alleles matching).

## Table S10: Detected virulence genes

| **Isolate** | **ST** | ***bceT*** | ***bpsD*** | ***bpsE*** | ***bpsF*** | ***bpsH*** | ***cerA*** | ***cerB*** | ***cesA*** | ***cesB*** | ***cesC*** | ***cesD*** | ***clo*** | ***cytK2*** | ***entA*** | ***entFM*** | ***hblA*** | ***hblB*** | ***hblC*** | ***hblD*** | ***hlyR*** | ***inhA1*** | ***inhA2*** | ***nheA*** | ***nheB*** | ***nheC*** | ***plcA*** | ***plcB*** | ***plcR*** | ***sph*** |
| --- | --- | --- | --- | --- | --- | --- | --- | --- | --- | --- | --- | --- | --- | --- | --- | --- | --- | --- | --- | --- | --- | --- | --- | --- | --- | --- | --- | --- | --- | --- |
| 71-1 | 24 | 1 | 1 | 1 | 1 | 1 | 1 | 1 | 0 | 0 | 0 | 0 | 1 | 1 | 1 | 1 | 1 | 1 | 1 | 1 | 1 | 1 | 1 | 1 | 1 | 1 | 1 | 1 | 1 | 1 |
| 71-2 | 24 | 1 | 1 | 1 | 1 | 1 | 1 | 1 | 0 | 0 | 0 | 0 | 1 | 1 | 1 | 1 | 1 | 1 | 1 | 1 | 1 | 1 | 1 | 1 | 1 | 1 | 1 | 1 | 1 | 1 |
| 55-1 | 26 | 0 | 0 | 1 | 1 | 1 | 1 | 1 | 1 | 1 | 1 | 1 | 1 | 0 | 1 | 1 | 0 | 0 | 0 | 0 | 0 | 1 | 1 | 1 | 1 | 1 | 1 | 1 | 1 | 1 |
| 05-1 | 32 | 0 | 0 | 1 | 1 | 1 | 1 | 1 | 0 | 0 | 0 | 0 | 1 | 1 | 1 | 1 | 0 | 0 | 0 | 0 | 0 | 1 | 1 | 1 | 1 | 1 | 1 | 1 | 1 | 1 |
| 05-2 | 32 | 0 | 0 | 1 | 1 | 1 | 1 | 1 | 0 | 0 | 0 | 0 | 1 | 1 | 1 | 1 | 0 | 0 | 0 | 0 | 0 | 1 | 1 | 1 | 1 | 1 | 1 | 1 | 1 | 1 |
| 26-1 | 32 | 0 | 0 | 1 | 1 | 1 | 1 | 1 | 0 | 0 | 0 | 0 | 1 | 1 | 1 | 1 | 0 | 0 | 0 | 0 | 0 | 1 | 1 | 1 | 1 | 1 | 1 | 1 | 1 | 1 |
| 26-2 | 32 | 0 | 0 | 1 | 1 | 1 | 1 | 1 | 0 | 0 | 0 | 0 | 1 | 1 | 1 | 1 | 0 | 0 | 0 | 0 | 0 | 1 | 1 | 1 | 1 | 1 | 1 | 1 | 1 | 1 |
| 74-1 | 32 | 0 | 0 | 1 | 1 | 1 | 1 | 1 | 0 | 0 | 0 | 0 | 1 | 1 | 1 | 1 | 0 | 0 | 0 | 0 | 0 | 1 | 1 | 1 | 1 | 1 | 1 | 1 | 1 | 1 |
| 74-2 | 32 | 0 | 0 | 1 | 1 | 1 | 1 | 1 | 0 | 0 | 0 | 0 | 1 | 1 | 1 | 1 | 0 | 0 | 0 | 0 | 0 | 1 | 1 | 1 | 1 | 1 | 1 | 1 | 1 | 1 |
| 75-1 | 32 | 0 | 0 | 1 | 1 | 1 | 1 | 1 | 0 | 0 | 0 | 0 | 1 | 1 | 1 | 1 | 0 | 0 | 0 | 0 | 0 | 1 | 1 | 1 | 1 | 1 | 1 | 1 | 1 | 1 |
| 75-2 | 32 | 0 | 0 | 1 | 1 | 1 | 1 | 1 | 0 | 0 | 0 | 0 | 1 | 1 | 1 | 1 | 0 | 0 | 0 | 0 | 0 | 1 | 1 | 1 | 1 | 1 | 1 | 1 | 1 | 1 |
| 61-1 | 164 | 0 | 0 | 1 | 1 | 1 | 1 | 1 | 0 | 0 | 0 | 0 | 1 | 0 | 1 | 1 | 0 | 0 | 0 | 0 | 0 | 1 | 1 | 1 | 1 | 1 | 1 | 1 | 1 | 1 |
| 19-2 | 165 | 0 | 0 | 1 | 1 | 1 | 1 | 1 | 1 | 1 | 1 | 1 | 1 | 0 | 1 | 1 | 0 | 0 | 0 | 0 | 0 | 1 | 1 | 1 | 1 | 1 | 1 | 1 | 1 | 1 |
| 37-1 | 165 | 0 | 0 | 1 | 1 | 1 | 1 | 1 | 0 | 0 | 0 | 0 | 1 | 0 | 1 | 1 | 0 | 0 | 0 | 0 | 0 | 1 | 1 | 1 | 1 | 1 | 1 | 1 | 1 | 1 |
| 37-2 | 165 | 0 | 0 | 1 | 1 | 1 | 1 | 1 | 0 | 0 | 0 | 0 | 1 | 0 | 1 | 1 | 0 | 0 | 0 | 0 | 0 | 1 | 1 | 1 | 1 | 1 | 1 | 1 | 1 | 1 |
| 41-1 | 165 | 0 | 0 | 1 | 1 | 1 | 1 | 1 | 1 | 1 | 1 | 1 | 1 | 0 | 1 | 1 | 0 | 0 | 0 | 0 | 0 | 1 | 1 | 1 | 1 | 1 | 1 | 1 | 1 | 1 |
| 41-2 | 165 | 0 | 0 | 1 | 1 | 1 | 1 | 1 | 1 | 1 | 1 | 1 | 1 | 0 | 1 | 1 | 0 | 0 | 0 | 0 | 0 | 1 | 1 | 1 | 1 | 1 | 1 | 1 | 1 | 1 |
| 42-1 | 165 | 0 | 0 | 1 | 1 | 1 | 1 | 1 | 0 | 0 | 0 | 0 | 1 | 0 | 1 | 1 | 0 | 0 | 0 | 0 | 0 | 1 | 1 | 1 | 1 | 1 | 1 | 1 | 1 | 1 |
| 42-2 | 165 | 0 | 0 | 1 | 1 | 1 | 1 | 1 | 1 | 1 | 1 | 1 | 1 | 0 | 1 | 1 | 0 | 0 | 0 | 0 | 0 | 1 | 1 | 1 | 1 | 1 | 1 | 1 | 1 | 1 |
| 51-1 | 165 | 0 | 0 | 1 | 1 | 1 | 1 | 1 | 1 | 1 | 1 | 1 | 1 | 0 | 1 | 1 | 0 | 0 | 0 | 0 | 0 | 1 | 1 | 1 | 1 | 1 | 1 | 1 | 1 | 1 |
| 51-2 | 165 | 0 | 0 | 1 | 1 | 1 | 1 | 1 | 1 | 1 | 1 | 1 | 1 | 0 | 1 | 1 | 0 | 0 | 0 | 0 | 0 | 1 | 1 | 1 | 1 | 1 | 1 | 1 | 1 | 1 |
| 64-2 | 165 | 0 | 0 | 1 | 1 | 1 | 1 | 1 | 1 | 1 | 1 | 1 | 1 | 0 | 1 | 1 | 0 | 0 | 0 | 0 | 0 | 1 | 1 | 1 | 1 | 1 | 1 | 1 | 1 | 1 |
| 35-1 | 205 | 0 | 0 | 1 | 1 | 1 | 1 | 1 | 0 | 0 | 0 | 0 | 1 | 1 | 1 | 1 | 0 | 0 | 0 | 0 | 0 | 1 | 1 | 1 | 1 | 1 | 1 | 1 | 1 | 1 |
| 35-2 | 205 | 0 | 0 | 1 | 1 | 1 | 1 | 1 | 0 | 0 | 0 | 0 | 1 | 1 | 1 | 1 | 0 | 0 | 0 | 0 | 0 | 1 | 1 | 1 | 1 | 1 | 1 | 1 | 1 | 1 |
| 63-1 | 205 | 0 | 0 | 1 | 1 | 1 | 1 | 1 | 0 | 0 | 0 | 0 | 1 | 1 | 1 | 1 | 0 | 0 | 0 | 0 | 0 | 1 | 1 | 1 | 1 | 1 | 1 | 1 | 1 | 1 |
| 63-2 | 205 | 0 | 0 | 1 | 1 | 1 | 1 | 1 | 0 | 0 | 0 | 0 | 1 | 1 | 1 | 1 | 0 | 0 | 0 | 0 | 0 | 1 | 1 | 1 | 1 | 1 | 1 | 1 | 1 | 1 |
| 19-1 | 266 | 0 | 0 | 1 | 1 | 1 | 1 | 1 | 0 | 0 | 0 | 0 | 1 | 0 | 1 | 1 | 0 | 0 | 0 | 0 | 0 | 1 | 1 | 1 | 1 | 1 | 1 | 1 | 1 | 1 |
| 55-2 | 266 | 0 | 0 | 1 | 1 | 1 | 1 | 1 | 0 | 0 | 0 | 0 | 1 | 0 | 1 | 1 | 0 | 0 | 0 | 0 | 0 | 1 | 1 | 1 | 1 | 1 | 1 | 1 | 1 | 1 |
| 59-1 | 266 | 0 | 0 | 1 | 1 | 1 | 1 | 1 | 0 | 0 | 0 | 0 | 1 | 0 | 1 | 1 | 0 | 0 | 0 | 0 | 0 | 1 | 1 | 1 | 1 | 1 | 1 | 1 | 1 | 1 |
| 64-1 | 266 | 0 | 0 | 1 | 1 | 1 | 1 | 1 | 0 | 0 | 0 | 0 | 1 | 0 | 1 | 1 | 0 | 0 | 0 | 0 | 0 | 1 | 1 | 1 | 1 | 1 | 1 | 1 | 1 | 1 |
| 44-1 | 1066 | 0 | 0 | 1 | 1 | 1 | 1 | 1 | 0 | 0 | 0 | 0 | 1 | 1 | 1 | 1 | 0 | 0 | 0 | 0 | 0 | 1 | 1 | 1 | 1 | 1 | 1 | 1 | 1 | 1 |
| 44-2 | 1066 | 0 | 0 | 1 | 1 | 1 | 1 | 1 | 0 | 0 | 0 | 0 | 1 | 1 | 1 | 1 | 0 | 0 | 0 | 0 | 0 | 1 | 1 | 1 | 1 | 1 | 1 | 1 | 1 | 1 |
| 59-2 | 1355 | 1 | 1 | 1 | 1 | 1 | 1 | 1 | 0 | 0 | 0 | 0 | 1 | 1 | 1 | 1 | 0 | 0 | 0 | 0 | 0 | 1 | 1 | 1 | 1 | 1 | 1 | 1 | 1 | 1 |
| 61-2 | 1443 | 0 | 0 | 1 | 1 | 1 | 1 | 1 | 0 | 0 | 0 | 0 | 1 | 0 | 1 | 1 | 0 | 0 | 0 | 0 | 0 | 1 | 1 | 1 | 1 | 1 | 1 | 1 | 1 | 1 |
| 07-1 | 2331 | 0 | 0 | 1 | 1 | 1 | 1 | 1 | 0 | 0 | 0 | 0 | 1 | 1 | 1 | 1 | 0 | 0 | 0 | 0 | 0 | 1 | 1 | 1 | 1 | 1 | 1 | 1 | 1 | 1 |
| 07-2 | 2331 | 0 | 0 | 1 | 1 | 1 | 1 | 1 | 0 | 0 | 0 | 0 | 1 | 1 | 1 | 1 | 0 | 0 | 0 | 0 | 0 | 1 | 1 | 1 | 1 | 1 | 1 | 1 | 1 | 1 |

The first column lists the isolate name, the remaining column lists the presence of the corresponding gene. A ‘0’ indicates absence of the gene, a ‘1’ indicates that the gene is present.

## Table S11: Detected AMR genes

| **isolate** | **ST** | ***bla*** | ***bla2*** | ***fosB*** | ***fosBx1*** | ***satA*** |
| --- | --- | --- | --- | --- | --- | --- |
| 71-1 | 24 | 1 | 1 | 0 | 1 | 0 |
| 71-2 | 24 | 1 | 1 | 0 | 1 | 0 |
| 55-1 | 26 | 1 | 1 | 1 | 0 | 0 |
| 05-1 | 32 | 0 | 1 | 1 | 0 | 1 |
| 05-2 | 32 | 0 | 1 | 1 | 0 | 1 |
| 26-1 | 32 | 0 | 1 | 1 | 0 | 1 |
| 26-2 | 32 | 0 | 1 | 1 | 0 | 1 |
| 74-1 | 32 | 0 | 1 | 0 | 0 | 1 |
| 74-2 | 32 | 0 | 1 | 0 | 0 | 1 |
| 75-1 | 32 | 0 | 1 | 0 | 0 | 1 |
| 75-2 | 32 | 0 | 1 | 0 | 0 | 1 |
| 61-1 | 164 | 1 | 1 | 1 | 0 | 0 |
| 19-2 | 165 | 1 | 1 | 1 | 0 | 0 |
| 37-1 | 165 | 1 | 1 | 1 | 0 | 0 |
| 37-2 | 165 | 1 | 1 | 1 | 0 | 0 |
| 41-1 | 165 | 1 | 1 | 1 | 0 | 0 |
| 41-2 | 165 | 1 | 1 | 1 | 0 | 0 |
| 42-1 | 165 | 1 | 1 | 1 | 0 | 0 |
| 42-2 | 165 | 1 | 1 | 1 | 0 | 0 |
| 51-1 | 165 | 1 | 1 | 1 | 0 | 0 |
| 51-2 | 165 | 1 | 1 | 1 | 0 | 0 |
| 64-2 | 165 | 1 | 1 | 1 | 0 | 0 |
| 35-1 | 205 | 1 | 1 | 1 | 0 | 0 |
| 35-2 | 205 | 1 | 1 | 1 | 0 | 0 |
| 63-1 | 205 | 1 | 1 | 1 | 0 | 0 |
| 63-2 | 205 | 1 | 1 | 1 | 0 | 0 |
| 19-1 | 266 | 1 | 1 | 1 | 0 | 0 |
| 55-2 | 266 | 1 | 1 | 1 | 0 | 0 |
| 59-1 | 266 | 1 | 1 | 1 | 0 | 0 |
| 64-1 | 266 | 1 | 1 | 1 | 0 | 0 |
| 44-1 | 1066 | 1 | 1 | 1 | 0 | 0 |
| 44-2 | 1066 | 1 | 1 | 1 | 0 | 0 |
| 59-2 | 1355 | 1 | 1 | 1 | 0 | 0 |
| 61-2 | 1443 | 1 | 1 | 1 | 0 | 1 |
| 07-1 | 2331 | 1 | 1 | 1 | 0 | 1 |
| 07-2 | 2331 | 1 | 1 | 1 | 0 | 1 |

The first column lists the isolate name, the remaining column lists the presence of the corresponding gene. A ‘0’ indicates absence, a ‘1’ indicates that the gene is present.

## Table S12: Plasmids detected by plasmidID

| **Isolate** | **ST** | **CP041977.1** | **CP041980.1** | **CP045535.1** | **NC_011771.1** | **NC_011776.1** | **NC_016773.1** | **NC_018494.1** | **NC_018885.1** | **NZ_AP022996.1** | **NZ_AP024509.1** | **NZ_CP004866.1** | **NZ_CP016363.1** | **NZ_CP023181.1** | **NZ_CP033794.1** | **NZ_CP036061.1** | **NZ_CP036144.1** | **NZ_CP043967.1** | **NZ_CP043968.1** | **NZ_CP045772.1** | **NZ_CP045773.1** | **NZ_CP045776.1*** | **NZ_CP047087.1** | **NZ_CP053978.1** | **NZ_CP053994.1** | **NZ_CP054814.1** | **NZ_CP064085.1** | **NZ_CP072768.1** |
| --- | --- | --- | --- | --- | --- | --- | --- | --- | --- | --- | --- | --- | --- | --- | --- | --- | --- | --- | --- | --- | --- | --- | --- | --- | --- | --- | --- | --- |
| 71-1 | 24 | 0 | 0 | 0 | 0 | 1 | 0 | 0 | 0 | 1 | 0 | 0 | 0 | 0 | 0 | 1 | 1 | 1 | 1 | 0 | 0 | 0 | 0 | 0 | 0 | 0 | 0 | 0 |
| 71-2 | 24 | 0 | 0 | 0 | 0 | 1 | 0 | 0 | 0 | 0 | 0 | 0 | 0 | 0 | 0 | 1 | 1 | 1 | 1 | 0 | 0 | 0 | 0 | 0 | 0 | 0 | 0 | 0 |
| 55-1 | 26 | 0 | 0 | 0 | 0 | 0 | 1 | 0 | 0 | 0 | 0 | 0 | 0 | 0 | 0 | 1 | 1 | 0 | 0 | 1 | 0 | 0 | 1 | 0 | 0 | 0 | 0 | 0 |
| 05-1 | 32 | 0 | 0 | 0 | 0 | 0 | 0 | 0 | 0 | 0 | 0 | 0 | 0 | 0 | 0 | 1 | 1 | 0 | 0 | 0 | 0 | 0 | 0 | 0 | 1 | 0 | 0 | 0 |
| 05-2 | 32 | 0 | 0 | 0 | 0 | 0 | 0 | 0 | 0 | 0 | 0 | 0 | 0 | 0 | 0 | 1 | 1 | 0 | 0 | 0 | 0 | 0 | 0 | 0 | 1 | 0 | 0 | 0 |
| 26-1 | 32 | 0 | 0 | 0 | 0 | 0 | 0 | 0 | 0 | 1 | 0 | 0 | 0 | 0 | 0 | 1 | 1 | 0 | 0 | 0 | 0 | 0 | 0 | 0 | 1 | 0 | 0 | 0 |
| 26-2 | 32 | 0 | 0 | 0 | 0 | 0 | 0 | 0 | 0 | 1 | 0 | 0 | 0 | 0 | 0 | 1 | 1 | 0 | 0 | 0 | 0 | 0 | 0 | 0 | 1 | 0 | 0 | 0 |
| 74-1 | 32 | 1 | 1 | 1 | 0 | 1 | 0 | 0 | 1 | 1 | 0 | 0 | 0 | 0 | 0 | 1 | 1 | 0 | 0 | 0 | 0 | 0 | 0 | 0 | 0 | 0 | 0 | 0 |
| 74-2 | 32 | 1 | 1 | 1 | 0 | 1 | 0 | 0 | 0 | 0 | 0 | 0 | 0 | 0 | 0 | 1 | 1 | 0 | 0 | 0 | 0 | 0 | 0 | 0 | 0 | 0 | 0 | 0 |
| 75-1 | 32 | 1 | 1 | 1 | 0 | 0 | 0 | 0 | 0 | 1 | 0 | 0 | 1 | 0 | 0 | 1 | 1 | 0 | 0 | 0 | 0 | 0 | 0 | 0 | 0 | 0 | 0 | 0 |
| 75-2 | 32 | 1 | 1 | 1 | 0 | 1 | 0 | 0 | 0 | 1 | 0 | 0 | 0 | 0 | 0 | 1 | 1 | 0 | 0 | 0 | 0 | 0 | 0 | 0 | 0 | 0 | 0 | 0 |
| 61-1 | 164 | 0 | 0 | 0 | 1 | 0 | 1 | 1 | 0 | 0 | 0 | 0 | 0 | 0 | 0 | 1 | 1 | 0 | 0 | 0 | 0 | 0 | 0 | 0 | 0 | 0 | 0 | 0 |
| 19-2 | 165 | 0 | 0 | 0 | 0 | 0 | 0 | 0 | 0 | 1 | 0 | 1 | 0 | 0 | 1 | 1 | 0 | 0 | 0 | 1 | 1 | 1 | 1 | 0 | 0 | 0 | 0 | 0 |
| 37-1 | 165 | 0 | 0 | 0 | 0 | 0 | 0 | 0 | 0 | 0 | 0 | 1 | 0 | 0 | 0 | 1 | 1 | 0 | 0 | 1 | 1 | 0 | 1 | 0 | 0 | 1 | 0 | 0 |
| 37-2 | 165 | 0 | 0 | 0 | 0 | 0 | 0 | 0 | 0 | 0 | 0 | 1 | 0 | 0 | 0 | 1 | 1 | 0 | 0 | 1 | 1 | 0 | 1 | 0 | 0 | 0 | 0 | 0 |
| 41-1 | 165 | 0 | 0 | 0 | 0 | 0 | 1 | 0 | 0 | 1 | 0 | 1 | 0 | 0 | 0 | 1 | 1 | 0 | 0 | 1 | 0 | 1 | 1 | 0 | 0 | 1 | 1 | 0 |
| 41-2 | 165 | 0 | 0 | 0 | 0 | 0 | 1 | 0 | 0 | 1 | 0 | 1 | 0 | 0 | 1 | 1 | 1 | 0 | 0 | 1 | 0 | 1 | 1 | 0 | 0 | 0 | 1 | 0 |
| 42-1 | 165 | 0 | 0 | 0 | 0 | 0 | 1 | 0 | 0 | 0 | 0 | 1 | 0 | 0 | 1 | 1 | 1 | 0 | 0 | 1 | 0 | 0 | 1 | 0 | 0 | 1 | 1 | 0 |
| 42-2 | 165 | 0 | 0 | 0 | 0 | 0 | 1 | 0 | 0 | 1 | 0 | 1 | 0 | 0 | 1 | 1 | 0 | 0 | 0 | 1 | 0 | 1 | 1 | 0 | 0 | 0 | 1 | 0 |
| 51-1 | 165 | 0 | 0 | 0 | 0 | 0 | 1 | 0 | 0 | 1 | 0 | 1 | 0 | 0 | 0 | 1 | 1 | 0 | 0 | 1 | 0 | 1 | 1 | 0 | 0 | 0 | 1 | 0 |
| 51-2 | 165 | 0 | 0 | 0 | 0 | 0 | 1 | 0 | 0 | 1 | 0 | 1 | 0 | 0 | 1 | 1 | 1 | 0 | 0 | 1 | 0 | 1 | 1 | 0 | 0 | 0 | 1 | 0 |
| 64-2 | 165 | 0 | 0 | 0 | 0 | 0 | 1 | 0 | 0 | 1 | 0 | 1 | 0 | 0 | 1 | 1 | 1 | 0 | 0 | 1 | 0 | 1 | 1 | 0 | 0 | 0 | 1 | 0 |
| 35-1 | 205 | 0 | 0 | 0 | 0 | 0 | 0 | 0 | 0 | 0 | 0 | 0 | 0 | 0 | 0 | 1 | 1 | 0 | 0 | 0 | 0 | 0 | 0 | 0 | 0 | 1 | 0 | 0 |
| 35-2 | 205 | 0 | 0 | 0 | 0 | 0 | 0 | 0 | 0 | 0 | 0 | 0 | 0 | 0 | 0 | 1 | 1 | 0 | 0 | 0 | 0 | 0 | 0 | 0 | 0 | 0 | 0 | 0 |
| 63-1 | 205 | 0 | 0 | 0 | 0 | 0 | 0 | 0 | 0 | 0 | 0 | 0 | 0 | 0 | 0 | 1 | 1 | 0 | 0 | 0 | 0 | 0 | 0 | 0 | 0 | 0 | 0 | 0 |
| 63-2 | 205 | 0 | 0 | 0 | 0 | 0 | 0 | 0 | 0 | 0 | 0 | 0 | 0 | 0 | 0 | 1 | 1 | 0 | 0 | 0 | 0 | 0 | 0 | 0 | 0 | 0 | 0 | 0 |
| 19-1 | 266 | 0 | 1 | 0 | 0 | 0 | 0 | 0 | 0 | 1 | 0 | 0 | 0 | 0 | 0 | 1 | 1 | 0 | 0 | 0 | 0 | 0 | 0 | 0 | 0 | 1 | 0 | 0 |
| 55-2 | 266 | 0 | 1 | 0 | 0 | 0 | 0 | 0 | 0 | 0 | 0 | 0 | 0 | 0 | 0 | 1 | 1 | 0 | 0 | 0 | 0 | 0 | 0 | 0 | 0 | 0 | 0 | 0 |
| 59-1 | 266 | 0 | 1 | 0 | 0 | 0 | 0 | 0 | 0 | 0 | 0 | 0 | 0 | 0 | 0 | 1 | 1 | 0 | 0 | 0 | 0 | 0 | 0 | 0 | 0 | 0 | 0 | 0 |
| 64-1 | 266 | 0 | 1 | 0 | 0 | 0 | 0 | 0 | 0 | 0 | 0 | 0 | 0 | 0 | 0 | 1 | 1 | 0 | 0 | 0 | 0 | 0 | 0 | 0 | 0 | 0 | 0 | 0 |
| 44-1 | 1066 | 0 | 1 | 0 | 0 | 0 | 0 | 0 | 0 | 1 | 0 | 0 | 0 | 1 | 0 | 1 | 1 | 0 | 0 | 1 | 0 | 0 | 0 | 1 | 0 | 0 | 0 | 0 |
| 44-2 | 1066 | 0 | 1 | 0 | 0 | 0 | 0 | 0 | 0 | 1 | 0 | 0 | 0 | 1 | 0 | 1 | 1 | 0 | 0 | 1 | 0 | 0 | 0 | 1 | 0 | 0 | 0 | 0 |
| 59-2 | 1355 | 0 | 0 | 0 | 1 | 0 | 0 | 0 | 0 | 0 | 0 | 0 | 1 | 0 | 0 | 1 | 1 | 0 | 0 | 0 | 0 | 0 | 0 | 0 | 0 | 0 | 0 | 1 |
| 61-2 | 1443 | 0 | 1 | 0 | 0 | 0 | 0 | 0 | 0 | 0 | 0 | 0 | 0 | 0 | 0 | 1 | 1 | 0 | 0 | 1 | 0 | 0 | 0 | 0 | 0 | 0 | 0 | 0 |
| 07-1 | 2331 | 0 | 0 | 0 | 0 | 0 | 0 | 0 | 0 | 0 | 1 | 0 | 0 | 0 | 0 | 1 | 1 | 0 | 0 | 0 | 0 | 0 | 0 | 0 | 0 | 0 | 0 | 0 |
| 07-2 | 2331 | 0 | 0 | 0 | 0 | 0 | 0 | 0 | 0 | 0 | 1 | 0 | 0 | 0 | 0 | 1 | 1 | 0 | 0 | 0 | 0 | 0 | 0 | 0 | 0 | 0 | 0 | 0 |

Plasmids detected by plasmidID, ‘1’ indicates presence (at least 80% covered), ‘0’ indicates absence of the corresponding plasmid. Notes: (*) The NZ_CP045776.1 plasmid contains the *ces* operon.

## Table S13: Pairwise SNP distances for group A (ST32)

| **Isolate** | **05-1** | **05-2** | **26-1** | **26-2** | **74-1** | **74-2** | **75-1** | **75-2** |
| --- | --- | --- | --- | --- | --- | --- | --- | --- |
| **05-1** | 0 | 0 | 1 | 1 | 90 | 69 | 91 | 90 |
| **05-2** | 0 | 0 | 1 | 1 | 90 | 69 | 91 | 90 |
| **26-1** | 1 | 1 | 0 | 0 | 88 | 66 | 89 | 88 |
| **26-2** | 1 | 1 | 0 | 0 | 89 | 68 | 90 | 89 |
| **74-1** | 90 | 90 | 88 | 89 | 0 | 32 | 0 | 1 |
| **74-2** | 69 | 69 | 66 | 68 | 32 | 0 | 31 | 30 |
| **75-1** | 91 | 91 | 89 | 90 | 0 | 31 | 0 | 1 |
| **75-2** | 90 | 90 | 88 | 89 | 1 | 30 | 1 | 0 |

This table lists the pairwise SNP distances between each pair of isolates. Note that the number of positions that are considered can be different for each comparison.

## Table S14: Pairwise SNP distances for group B (ST165)

|  | **19-2** | **37-1** | **37-2** | **41-1** | **41-2** | **42-1** | **42-2** | **51-1** | **51-2** | **64-2** |
| --- | --- | --- | --- | --- | --- | --- | --- | --- | --- | --- |
| **19-2** | 0 | 1 | 1 | 7 | 8 | 9 | 8 | 9 | 7 | 5 |
| **37-1** | 1 | 0 | 0 | 6 | 7 | 8 | 7 | 8 | 6 | 4 |
| **37-2** | 1 | 0 | 0 | 6 | 7 | 8 | 7 | 8 | 6 | 4 |
| **41-1** | 7 | 6 | 6 | 0 | 0 | 4 | 4 | 4 | 1 | 4 |
| **41-2** | 8 | 7 | 7 | 0 | 0 | 5 | 5 | 5 | 1 | 5 |
| **42-1** | 9 | 8 | 8 | 4 | 5 | 0 | 6 | 6 | 4 | 6 |
| **42-2** | 8 | 7 | 7 | 4 | 5 | 6 | 0 | 4 | 4 | 5 |
| **51-1** | 9 | 8 | 8 | 4 | 5 | 6 | 4 | 0 | 4 | 6 |
| **51-2** | 7 | 6 | 6 | 1 | 1 | 4 | 4 | 4 | 0 | 4 |
| **64-2** | 5 | 4 | 4 | 4 | 5 | 6 | 5 | 6 | 4 | 0 |

This table lists the pairwise SNP distances between the isolates. Note that the number of positions that are considered can be different for each comparison.

## Table S15: Pairwise SNP distances for group C (ST205)

| **Isolate** | **35-1** | **35-2** | **63-1** | **63-2** |
| --- | --- | --- | --- | --- |
| **35-1** | 0 | 0 | 3 | 3 |
| **35-2** | 0 | 0 | 3 | 3 |
| **63-1** | 3 | 3 | 0 | 0 |
| **63-2** | 3 | 3 | 0 | 0 |

This table lists the pairwise SNP distances between each pair of isolates. Note that the number of positions that are considered can be different for each comparison.

## Table S16: Pairwise SNP distances for group D (ST266)

| **Isolate** | **19-1** | **55-2** | **59-1** | **64-1** |
| --- | --- | --- | --- | --- |
| **19-1** | 0 | 2 | 0 | 27 |
| **55-2** | 2 | 0 | 2 | 26 |
| **59-1** | 0 | 2 | 0 | 24 |
| **64-1** | 27 | 26 | 24 | 0 |

This table lists the pairwise SNP distances between the isolates. Note that the number of positions that are considered can be different for each comparison.

# References

[1] C. Camacho *et al.*, “BLAST+: architecture and applications,” *BMC Bioinformatics*, vol. 10, no. 1, p. 421, Dec. 2009, doi: 10.1186/1471-2105-10-421.

[2] J. A. Wintersinger and J. D. Wasmuth, “Kablammo: an interactive, web-based BLAST results visualizer,” *Bioinformatics*, vol. 31, no. 8, pp. 1305–1306, Apr. 2015, doi: 10.1093/bioinformatics/btu808.

[3] V. Galata, T. Fehlmann, C. Backes, and A. Keller, “PLSDB: a resource of complete bacterial plasmids,” *Nucleic Acids Research*, vol. 47, no. D1, pp. D195–D202, Jan. 2019, doi: 10.1093/nar/gky1050.
